# Endocannabinoids, perioperative pain, and acetaminophen in patients undergoing total knee arthroplasty: a prospective cohort study

**DOI:** 10.1097/PR9.0000000000001369

**Published:** 2026-01-30

**Authors:** Nathan Clendenen, Anna Clendenen, Robert McClain, Garret Wheeler, Jost Klawitter, Uwe Christians, Steven R. Clendenen, Jelena Klawitter

**Affiliations:** a Department of Anesthesiology, School of Medicine, University of Colorado Anschutz Medical Campus, Aurora, CO, USA; b Department of Anesthesiology and Perioperative Medicine, Mayo Clinic, Jacksonville, FL, USA

**Keywords:** Endocannabinoid system, Osteoarthritis, Pain, Inflammation, Total knee arthroplasty

## Abstract

**Introduction:**

Total knee arthroplasty (TKA) for knee osteoarthritis (OA) provides pain relief and functional improvement for many patients. The endocannabinoid system (ECS) regulates pain and inflammation through central and peripheral mechanisms and may be affected by acetaminophen. There is limited information on how surgery and perioperative care affect the ECS.

**Objective:**

We designed this study to examine the effect of surgery and acetaminophen on the ECS and determine whether perioperative endocannabinoids were associated with pain resolution after surgery.

**Methods:**

Forty adult patients with OA undergoing TKA were enrolled in a prospective observational cohort study and provided preoperative cerebrospinal fluid (CSF) and blood samples at 3 time points (before surgery, after intravenous acetaminophen, and 24 hours postoperative). Defense and Veterans Pain Rating Scale scores at rest and with activity were recorded in the preoperative holding area, in the operating room after intravenous acetaminophen before spinal anesthesia, and 24 to 48 hours postoperatively. Cerebrospinal fluid and plasma endocannabinoids were quantified using a validated liquid chromatography/mass spectrometry assay. We analyzed the data using linear regression models with sex, age, and body mass index as covariates.

**Results:**

Patients undergoing TKA have higher CSF and plasma concentrations of N-acylethanolamines anandamide and its congeners compared with control subjects. Patients with higher pain scores had lower CSF anandamide levels before and after surgery and higher levels of 2-arachidonoylglycerol before surgery but not after.

**Conclusion:**

Chronic pain, acetaminophen, and surgery are associated with endogenous endocannabinoid levels and may present an opportunity for developing targeted nonopioid perioperative analgesia after TKA.

## Abstract 2

Supplemental Digital Content is Available in the Text.

Patients with osteoarthritis have increased anandamide before surgery which decreases after surgery. Postoperative anandamide levels are associated with long-term pain resolution after surgery.

## 1. Introduction

Osteoarthritis (OA) is the most common form of arthritis in adults affecting 8% of the global population^14^ and is the leading cause of chronic pain and long-term disability resulting in substantial health expenditure.^36^ Total knee arthroplasty (TKA) is effective for end-stage knee OA and provides pain relief and functional improvement for patients.^55^ Unfortunately, approximately 15% to 30% of patients develop chronic postoperative pain, defined as pain that lasts for at least 3 months after surgery.^48^ There is conflicting evidence on patient factors associated with pain perseverance such as body mass index (BMI), diabetes, and dyslipidemia; however, it remains unknown why many patients have persistent pain after TKA.^31,35^

Osteoarthritis pain is multifactorial, and a high percentage of patients with OA have low-grade chronic inflammation^47^ with elevation of proinflammatory cytokines and prostaglandins.^4,21,52^ Preclinical evidence suggests that proinflammatory mediators sensitize the peripheral and central nerves to enhance pain.^20^ A recent study linked preoperative inflammatory profiles to chronic postoperative pain after TKA.^15^ However, there is still a limited understanding of factors associated with worse pain outcomes.

Previous work identified the endocannabinoid system (ECS) as a key endogenous system regulating pain sensation, with modulatory action on all stages of pain processing pathways.^6,7,53^ Azim et al.^2^ reported that higher preoperative levels of 2-arachidonyl glyceryl ether (2-AG) before TKA in cerebrospinal fluid (CSF) and synovial fluid were associated with higher levels of pain and increased opioid requirements after surgery. Perioperative acetaminophen is a key therapeutic for multimodal analgesia after TKA,^12^ and the endocannabinoid system is necessary for acetaminophen-induced analgesia in animal models.^29,33,43^ It is unknown whether endogenous endocannabinoid levels change in response to acetaminophen in humans and whether perioperative endocannabinoid levels are associated with pain resolution after surgery.

In this study, we completed a prospective observational cohort study to evaluate plasma and CSF endocannabinoid levels measured by mass spectrometry to (1) compare differences between patients with knee OA and healthy controls, (2) to investigate differences in preoperative endocannabinoid levels of patients with knee OA, (3) determine the association between endocannabinoid levels and the development of postoperative pain, and (4) measure the impact of acetaminophen on the endocannabinoid system and postoperative pain. The purpose of this study was to test the hypothesis that exposure to acetaminophen before surgery modulates endocannabinoid levels and that perioperative endocannabinoid levels are associated with pain resolution after TKA.

We further anticipated that patients with OA present with higher circulating and CSF levels of anandamide (AEA) and 2-AG as compared with healthy individuals and that this dysregulation will be more pronounced in patients with prolonged postoperative pain. Overall, it was our goal to identify an endocannabinoid signature that would stratify patients more prone to developing postoperative pain and inform future studies to help individualize postsurgery pain management strategies.

## 2. Materials and methods

### 2.1. Study design

The Mayo Clinic institutional review board approved the study protocol (#17-003913), and written consent was obtained from each patient before enrollment. Study enrollment started in May 2018 and finished in September 2019 with the last follow-up contact in November 2020. We used a crossover observational study design to reduce bias with each patient serving as their own control to determine the effect of acetaminophen and TKA on their endocannabinoid levels. The inclusion criteria were patients scheduled for elective total knee arthroplasty with spinal anesthesia aged 18 years or older and an American Society of Anesthesiologists physical status of I, II, or III. The exclusion criteria were pregnancy, patients with contraindications to acetaminophen, patients with a risk of delayed gastric emptying due to diabetes with gastroparesis, patients on anticoagulation therapy, and patients with a history of lumbar spine surgery. Forty eligible TKA patients enrolled for this study were scheduled for elective unilateral TKA under spinal anesthesia and femoral nerve block as per the standard-of-care clinical protocol at the Mayo Clinic Jacksonville. Patients refrained from taking nonsteroidal anti-inflammatory drugs for 7 days before surgery. Patients received 1 gram of intravenous acetaminophen in the holding area 1 hour before transfer to the operating room. Celecoxib and gabapentin were administered in the postoperative anesthesia care unit.

### 2.2. Study variables and sample collection

Before anesthesia and surgery, the patients underwent assessment of pain status using the Defense and Veterans Pain Rating Scale (DVPRS) with 0 = no pain and 10 = worst possible pain. Knee pain was evaluated at rest and with activity (flexion and extension of the knee while sitting at the edge of the bed) at baseline, 24 to 48 hours by study personnel (S.R.C., R.M., A.C.) during routine pain rounds, and by phone survey administered by the study research nurse (A.C.) at approximately 7 days, 90 days, 180 days, and 365 days after surgery. Knee pain evaluation was not standardized in relation to the timing of analgesia medication administration. Additional functional items included the effect of pain on patients' level of activity, stress, sleep, mood, and overall satisfaction with surgery. Whole blood was collected from a peripheral vein into an EDTA vacutainer, placed immediately on ice, and centrifuged at 3,000 RCF for 10 minutes at 4°C to isolate platelet-poor plasma samples that were stored at −80°C until to analysis. Cerebrospinal fluid was collected into a sterile cryovial at the time of intrathecal access before spinal anesthetic administration and immediately placed on ice in an opaque bag and stored at −80°C until analysis. Plasma samples were collected before intravenous acetaminophen administration, 1 hour after acetaminophen, and 24 to 48 hours after surgery. Cerebrospinal fluid samples were collected in the operating room before administration of the spinal anesthetic. Confounders were selected a priori as potential covariates. These included age, sex, race, BMI, and pain medication use before surgery.

### 2.3. Quantitation of endocannabinoids and acetaminophen by liquid chromatography/tandem mass spectrometry

Levels of 2-AG, 2-AGE (2-arachidonyl glyceryl ether, noladin ether), docosatetraenoylethanolamide (DEA), linoleoylethanolamide (LEA), NADA (N-arachidonoyl dopamine), oleoylethanolamide (OEA), palmitoylethanolamide (PEA) and stearoylethanolamide (SEA) in CSF and EDTA plasma were measured using a one-step extraction followed by online trapping high-performance liquid chromatography–tandem mass spectrometry. The assay was validated in human plasma and intrarun and inter-run accuracies and precisions as well as matrix effects, recoveries, and sample stabilities had previously been reported.^50^ An abbreviated validation was performed for the analysis of CSF samples to ensure that the assay met specifications in this matrix.

In brief, to 200-μL aliquots of calibrator, quality control, or study samples, 800 μL of acetonitrile containing the internal standards (2 ng/mL for AEA-d5, 2-AG-d5, PEA-d4, LEA-d4, and OEA-d4, 8 ng/mL for d3-SEA, and 10 ng/mL for d8-NADA) were added. Samples were vortexed for 10 minutes and then centrifuged at 25,000*g* and 4°C for 10 minutes. Seven hundred and fifty (750) μL of the supernatant was transferred into high-performance liquid chromatography (HPLC) vials, 450 μL of 0.1% formic acid in water was added, and the extracts were vortexed. The sample extracts were placed into the HPLC autosampler for subsequent analysis. For further sample cleanup, online trapping was used; analytes were back-flushed using an HPLC switching valve onto the analytical column (Poroshell 120 EC-C18 2.7 μm, 4.6 × 50 mm, Agilent Technologies, Santa Clara, CA). A gradient of 0.1% formic acid in HPLC-MS grade water (Solvent A) and 0.1% formic acid in acetonitrile (Solvent B) was used for chromatographic separation. The HPLC system was interfaced with an API6500 QTRAP system (ABSciex, Foster City, CA) through a turbo-V ion source operated in the positive atmospheric pressure chemical ionization mode. The mass spectrometer was operated in the positive multiple reaction monitoring mode, and quantification of the ECs was performed using ABSciex Multiquant software version 3.0.^42^

Quantification of acetaminophen in plasma and CSF was performed using a validated liquid chromatography/tandem mass spectrometry assay.^51^

### 2.4. Statistical analyses

We designed the study with a sample size of N = 40 to provide 80% power to detect a 20% difference in the analyte levels between time points with ɑ = 0.05 assuming a 10% drop out rate. The study design was registered with the internal review board before enrollment. The study was not powered to detect differences between patients enrolled in the cohort and healthy control samples analyzed for reference. All patients completed the in-hospital time points with sample collection and postoperative assessment. Plasma sampling was complete at the 3 time points (120 samples), and CSF was collected for 39/40 patients (97.5%). We considered the outpatient time point assessment exploratory and did not adjust for missing data or for loss to follow-up after hospital discharge. Baseline characteristics are summarized and presented as mean ± standard deviation (normally distributed) or median with interquartile range (skewed) for continuous variables and number and percentage for categorical variables. We used the Shapiro–Wilk test to test for normality. Data those failed normality assessment were ln-transformed to reduce skewness. We used a 1-way ANOVA with post hoc Tukey test to assess for differences in endocannabinoid levels between healthy controls and patients with OA.

For the perioperative study, we used linear mixed models to test for an association between patient characteristics, clinical pain metrics, and endocannabinoids at baseline and on postoperative day 1 (POD1). We modelled DVPRS pain scores as the dependent variables and age, BMI, and endocannabinoids as continuous variables with fixed effects. We also assessed sex and any use of pain medication at baseline as fixed effects. Specific medications patients used before surgery included acetaminophen, celecoxib, fentanyl, gabapentin, hydromorphone, ibuprofen, ketorolac, meloxicam, naproxen, oxycodone, tramadol, acetaminophen with codeine, hydrocodone, or cannabidiol (CBD) oil. We did not assess the association between exposure to specific medications before surgery. We determined the association between endocannabinoid levels and exposure to intravenous acetaminophen and TKA with a linear mixed model assessing patient variability as a random effect and time point, age, sex, and BMI as fixed effects.

We considered a *P* < 0.05 statistically significant for all analyses.

In an exploratory analysis, we calculated the Spearman rank correlation coefficient between the study variables to identify possible associations between endocannabinoids, sex, age, and clinical outcomes by time point. We report the crude and false discovery rate adjusted *P* values for context. We used the Benjamini–Hochberg method to control for the family-wise error rate. Statistical analyses were performed using SPSS statistics (version 27, IBM, Armonk, NY) and JMP Pro (version 17, SAS Institute, Cary, NC).

## 3. Results

### 3.1. Subjects

**Figure 1.**
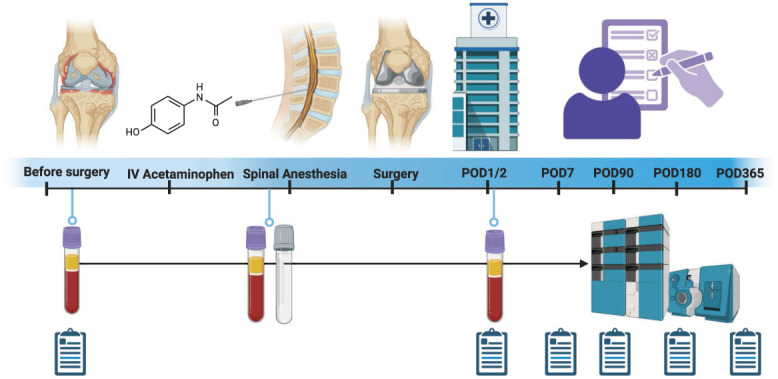
Schematic of the study timeline. Patients were consented before surgery and provided a baseline plasma sample and pain assessment. Next patients received intravenous acetaminophen followed by paired cerebrospinal fluid and plasma sampling immediately before spinal anesthesia and total knee arthroplasty. Plasma was sampled 24 hours after surgery with in-hospital pain assessment. Pain outcomes were assessed at baseline before surgery, postoperative days 1, 7, 90, 180, and 365 days after. Plasma and CSF samples were analyzed with LC-MS/MS. CSF, cerebrospinal fluid; LC-MS/MS, liquid chromatography/tandem mass spectrometry.

A schematic of the study timeline, sample collection, and clinical follow-up is presented in Figure 1. Demographic information of male and female patients with OA presenting for TKA surgery is summarized in Table 1. The mean preoperative pain scores were 2.3 ± 2.4 at rest and 6.2 ± 2.4 with movement. The mean pain scores on postoperative day 1 were 2.6 ± 2.3 at rest and 4.2 ± 2.8 with movement. Most patients used medications for knee pain before surgery (28/40, 70%) and 1 patient used CBD oil (Table 1). The mean follow-up time was 268 ± 153 days with 22/40 (55%) completing 1 year follow-up and 30/40 (75%) completing 6 months of follow-up. Overall 29.6 patient years (74%) of the planned 40 patient years of follow-up was available for analysis.

**Table 1:** Baseline cohort characteristics.

Characteristic	Patient cohort (n = 40)
Sex	27 female (68%); 13 male (33%)
Age (y)	68.7 ± 13.5
Race	4 Black (10%), 36 White (90%)
BMI (kg/m^2^)	32.7 ± 7.3
Medication use for knee pain before surgery	28 yes (70%), 12 no (30%)
DVPRS at rest: preop; day 1; day 7	2 (0, 3.5); 2.5 (0.75, 4); 3 (1, 4)
DVPRS with movement: preop; day 1; day 7	6 (4, 8); 4 (2, 5.5); 4 (2, 6)
Effect of pain on activity: preop; day 1; day 7	6 (4.5, 8); 5.5 (0.75, 8); 4 (0, 5)
Effect of pain on stress: preop; day 1; day 7	4 (0, 5); 0 (0, 1.5); 0 (0, 2.75)
Effect of pain on sleep: preop; day 1; day 7	4 (1.5, 6); 3 (0, 7); 0 (0, 5)
Effect of pain on mood: preop; day 1; day 7	3 (0, 5); 0 (0, 2); 0 (0, 3.75)

Data are presented as either means ± standard error of the mean or as medians with the interquartile range.

BMI, body mass index; DVPRS, Defense and Veterans Pain Rating Scale (Min: 0, Max: 10).

### 3.2. Cerebrospinal fluid and plasma levels of endocannabinoids in patients with osteoarthritis vs control subjects

In CSF collected from 50 controls (23 male, 27 female, 58.8 ± 7.8 years of age without OA, obtained from Precision Med, BioIVT, Westbury, NY), we were able to detect 2-AG, AEA, LEA OEA, and PEA in most, but not all samples (Table 2). The major endocannabinoids AEA and 2-AG were quantifiable in CSF from 17 and 38 control subjects, respectively (Table 2). Interestingly, 2-AG levels were not different between the control subjects and patients with OA (N = 39, Table 2). Anandamide, docosatetraenoylethanolamide (only quantifiable in patients with OA), LEA, and OEA were all significantly higher in the CSF of patients with OA vs controls (*P* < 0.001), whereas NADA concentrations were lower (*P* < 0.05) (Table 2). Noladin ether (2-AGE) was only present in CSF of patients with OA (Table 2).

**Table 2:** Endocannabinoids in plasma and cerebrospinal fluid.

Endocannabinoid levels in plasma		
Analyte	Patient cohort (n = 40)	Healthy controls (n = 100)^28^
2-LG (ng/mL)	3.29 (2.31, 7.04)*	7.26 [5.34, 10.2]
2-AG (ng/mL)	4.09 (2.62, 5.72)	3.81 [2.08, 6.10]
AEA (ng/mL)	2.23 ± 0.19***	0.77 [0.59, 0.99]
DEA (ng/mL)	0.29 ± 0.02	0.20 [0.12, 0.30]
LEA (pg/mL)	3.68 ± 0.30***	1.41 [1.08, 1.72]
OEA (pg/mL)	7.87 (4.87, 11.1)***	3.59 [2.91, 4.77]
PEA (pg/mL)	7.60 (5.01, 12.2)*	4.57 [3.51, 5.78]
SEA (pg/mL)	7.11 (5.92, 10.0)***	1.10 [0.89, 1.38]

Endocannabinoid levels in CSF		
Analyte	Patient cohort (n = 39)	Healthy controls (n = 50)
2-AG (pg/mL)	17.8 ± 1.52	20.3 ± 2.75 BLOQ, n = 12
2-AGE (pg/mL)	70.0 ± 8.38	—
AEA (pg/mL)	9.67 ± 0.52***	0.47 ± 0.11 BLOQ, n = 33
DEA (pg/mL)	4.34 (2.88, 5.49)	—
LEA (pg/mL)	14.4 ± 0.47***	8.41 ± 1.09 BLOQ, n = 11
NADA (pg/mL)	62.0 (57.4, 70.2)*	80.9 (58.7, 93.4)
OEA (pg/mL)	37.3 (30.6, 44.8)***	6.77 (4.58, 11.1) BLOQ, n = 14
PEA (pg/mL)	43.7 ± 0.98	67.1 ± 8.57 BLOQ, n = 12

Plasma samples were collected from healthy individuals (n = 100, published in Ref. 28) and patients with osteoarthritis undergoing TKA at baseline (n = 40 samples). Data are presented means ± standard error of the mean. Significance levels (as determined by ANOVA in combination with the Tukey post hoc test): *P < 0.05, ***P < 0.001. CSF samples were collected from heathy individuals without OA (n = 50, samples were purchased from BioIVT, Westbury, NY) and patients with OA undergoing TKA at baseline (n = 39).

In CSF samples (purchased from BioIVT, Westbury, NY) from 50 individuals without osteoarthritis, 2-AG was below the limit of quantitation in 12 subjects, AEA in 33, LEA in 11, OEA in 14, and PEA in 12 subjects. Significance levels (as determined by ANOVA in combination with the Tukey post hoc test): *P < 0.05, ***P < 0.001.

2-AG, 2-arachidonoyl glycerol; 2-AGE, 2-arachidonyl glyceryl ether/noladin ether; AEA, anandamide; BLOQ, bellow limit of quantitation; BMI, body mass index; CSF, cerebrospinal fluid; DEA, docosatetraenoylethanolamide; LEA, linoleoylethanolamide; n.d., not detectable; NADA, N-arachidonoyl dopamine; OA, osteoarthritis; OEA, oleoylethanolamide; PEA, palmitoylethanolamide; SEA, stearoylethanolamide; TKA, total knee arthroplasty.

Previously, we had established plasma endocannabinoid levels in healthy individuals (n = 100, 60 male [60%], 40 female [40%], age 41.0 years [30.0, 48.8]).^24^ Compared with healthy individuals, patients with OA presented with significantly higher levels of AEA and its congeners LEA, OEA, PEA, and SEA (Table 2).

Acetaminophen levels were measured in collected plasma and CSF samples. In CSF, the median acetaminophen concentration was 651 ng/mL (IQR: LLOQ, 1702 ng/mL), whereas the corresponding median plasma concentration was 2690 ng/mL (IQR: 1510, 5405 ng/mL).

### 3.3. Association between plasma and cerebrospinal fluid endocannabinoids with pre and postoperative pain

**Figure 2.**
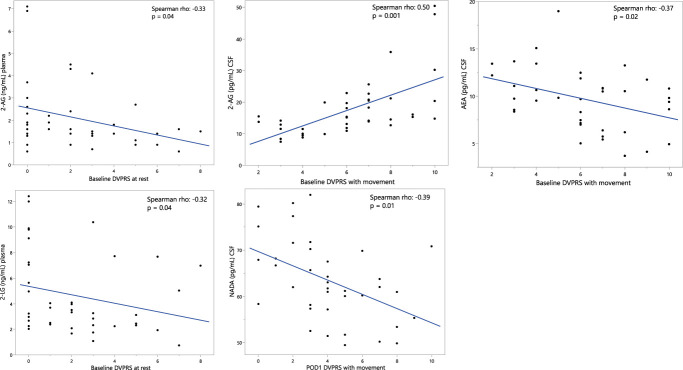
Significant correlations between analyte levels and pain at rest and with movement measured by the DVPRS pain scale at baseline or POD1 as indicated on the x axis of the figure. DVPRS, Defense and Veterans Pain Rating Scale; POD1, postoperative day 1.

In a crude analysis of the association between plasma and CSF endocannabinoid levels and pain, we observed significant correlations between 2-AG, 2-linoleoylglycerol (2-LG), AEA, and NADA and the DVPRS pain scores (Fig. 2). In an adjusted analysis controlling for age, sex, BMI, and medication use, preoperative CSF levels of 2-AG were independently associated with preoperative DVPRS with movement and CSF levels of AEA were inversely independently associated with preoperative DVPRS with movement (Table 3). We also evaluated the association of preoperative CSF endocannabinoids with postoperative pain outcomes on postoperative day 1, day 7, and at the end of follow-up (pain resolved: yes or no). However, none of the presurgical levels of CSF endocannabinoids were associated with the pain experienced 7 days or longer after surgery.

**Table 3:** Significant results from the adjusted analysis testing for associations between analytes and pain, time point, and interindividual variability.

Analyte	Time point	Estimate	Std error	Prob > t	95% lower	95% upper
Endocannabinoids in the CSF associated with pain during movement measured by the DVPRS						
2-AG CSF	Baseline	0.14	0.04	0.007	0.07	0.22
AEA CSF	Baseline	−0.26	0.11	0.03	−0.49	−0.02
Endocannabinoids in plasma with a statistically significant difference between time points						
PEA	APAP to baseline	2.59	1.29	0.047	0.03	5.15
SEA	APAP to baseline	2.21	1.05	0.04	0.11	4.31
2-AG	POD1 to APAP	0.76	0.37	0.046	0.02	1.5
AEA	POD1 to APAP	−0.94	0.28	0.001	−1.5	−0.39
LEA	POD1 to APAP	−1.16	0.52	0.03	−2.2	−0.13
OEA	POD1 to APAP	−3.16	1.20	0.01	−5.55	−0.77
PEA	POD1 to APAP	−2.80	1.31	0.04	−5.40	−0.20
2-LG	POD1 to APAP	2.76	1.33	0.045	0.07	5.5
AEA	POD1 to APAP	−0.54	0.24	0.03	−1.04	−0.04

Analyte	Time point	Estimate	Std error	Wald *P*	95% lower	95% upper
Endocannabinoids in plasma with a statistically significant difference due to intersubject variability when controlling for time point, age, sex, and BMI						
AEA	All	0.54	0.27	0.047	0.006	1.1
DEA	All	11,978	5,333	0.03	1,524	22,431
LEA	All	2.1	0.97	0.03	0.18	3.96

2-AG, 2-arachidonoyl glycerol; 2-LG, 2-linoleoylglycerol; AEA, anandamide; APAP, post-IV acetaminophen time point; BMI, body mass index; CSF, cerebrospinal fluid; DEA, docosatetraenoylethanolamide; DVPRS, Defense And Veterans Pain Rating Scale; LEA, linoleoylethanolamide; OEA, oleoylethanolamide; PEA, palmitoylethanolamide; POD1, postoperative day 1; SEA, stearoylethanolamide.

### 3.4. Time-dependent changes of plasma endocannabinoids during the perioperative period

**Figure 3.**
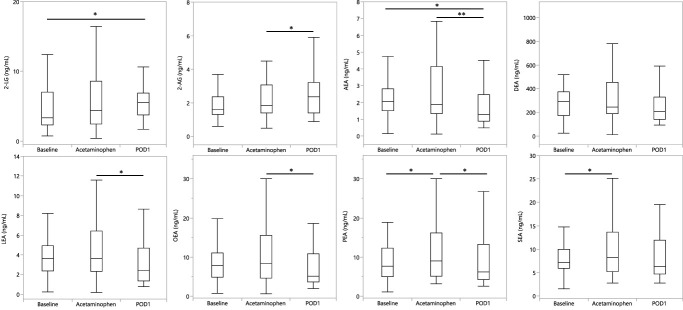
Effect of acetaminophen dosing and surgery on plasma endocannabinoids levels over time. Results are presented as a box plot displaying the minimum, maximum, median, and interquartile range and the significant level is from the analysis of the mixed-effect models controlling for age, sex, BMI, and subject. 2-AG, 2-arachidonoyl glycerol; 2-LG, 2-linoleoylglycerol; AEA, anandamide; BMI, body mass index; DEA, docosatetraenoylethanolamide; LEA, linoleoylethanolamide; OEA, oleoylethanolamide; PEA, palmitoylethanolamide; SEA, stearoylethanolamide. Significance levels: *P < 0.05, **P < 0.01.

In a linear multivariable mixed model with time point and sex as fixed factors and age and BMI as covariates with subject variability modelled as a random effect, we observed significant independent differences in plasma endocannabinoids after exposure to intravenous acetaminophen and surgery. Palmitoylethanolamide and stearoylethanolamide increased after intravenous acetaminophen and 2-AG increased on POD1 while AEA, LEA, OEA, and PEA decreased on POD1 compared with the postacetaminophen time points before surgery. 2-LG and AEA were decreased on POD1 compared with the baseline preacetaminophen time point (Fig. 3 and Table 3). The complete study measurements are available in Supplementary Table 1, http://links.lww.com/PR9/A360.

### 3.5. Association of baseline plasma endocannabinoids with long-term pain (pre- and postoperatively)

We did not observe an association between baseline plasma levels and pain experienced with knee movement before surgery. We did not observe an association between pain scores and endocannabinoids during the acute postoperative period (24 hours up to 7 days). However, higher plasma AEA, LEA, and PEA levels at baseline were associated with better pain outcomes and pain resolution, as reported at the end of follow-up after surgery (AEA: B = 1.056, odds ratio = 2.87, 95% CI 1.06–7.77, *P* = 0.038; LEA: B = 0.665, odds ratio = 1.93, 95% CI 1.03–3.60, *P* = 0.040; PEA: B = 0.299, odds ratio = 1.35, 95% CI 1.05–1.74, *P* = 0.021).

### 3.6. Exploratory correlations analysis

We observed statistically significant intersubject variability in plasma AEA, DEA, and LEA over time (Table 3). We also observed a significant correlation between patient age in years and CSF levels of OEA and O-AEA and plasma levels of 2-LG (Supplementary Table 2, http://links.lww.com/PR9/A360). We did not observe any significant correlations between sex and CSF endocannabinoid levels; however, plasma SEA levels were significantly higher in women compared with men at baseline (8.8 ± 4.0 95% CI 7.2–10.3 vs 6.3 ± 2.1 95% CI 5.1–7.6, *P* = 0.03). We also observed significant correlations between plasma and CSF endocannabinoid levels and secondary pain outcomes (activity levels, sleep, mood, and stress at baseline and on POD1, Supplementary Table 2, http://links.lww.com/PR9/A360), although only correlations between different endocannabinoid levels and different pain assessments were significant after correction for multiple comparisons.

## 4. Discussion

### 4.1. Key findings

We observed an independent inverse association between AEA CSF levels and the DVPRS pain score with movement in patients with OA supporting the role of AEA as being antinociceptive.^26^ Similarly, Azim et al. demonstrated that patients who reported more severe postoperative pain directly after TKA surgery had higher preoperative levels of 2-AG in CSF and synovial fluid, suggesting that 2-AG may predispose patients to acute pain after surgery.^2^ Our results likewise demonstrated an independent association between increased 2-AG CSF levels and preoperative pain; however, we did not observe an association between 2-AG and postoperative pain, possibly due to the preemptive acetaminophen administration included as part of the standardized multimodal analgesia protocol for perioperative pain management after TKA in our study. We also did not observe an association between plasma endocannabinoids and preoperative pain scores. However, higher preoperative plasma levels of AEA were associated with less pain at the last follow-up time point after surgery. Future studies are necessary to clarify this association and determine whether modulation of AEA levels would be beneficial for promoting pain resolution after TKA. During the perioperative period, we observed an increase in plasma levels of N-acylethanolamine after acetaminophen treatment followed by a decline and return to baseline levels 24 to 48 hours after surgery. The increase in N-acylethanolamine after acetaminophen may be similar to the observation in animals that acetaminophen protects against hyperalgesia through modulation of the endocannabinoid system interactions with CB1R receptors in the brain to prevent acute and inflammatory pain.^29^

### 4.2. Endocannabinoids and pain after surgery

Total knee arthroplasty is a key treatment for end-stage knee OA. Although pain after surgery resolves in most patients, 15% to 30% of patients still have persistent pain 6 months after surgery that cannot be otherwise explained (eg, through infection or joint loosening).^39,49^ The ECS has been shown to play a significant role in regulating pain perception through its interaction with cannabinoid receptors and the transient receptor potential vanilloid 1 (TRPV1) channel.^17,44,54^ In animal models of OA, modulation of CB1R^25,34^ (through an increase in hippocampal AEA levels) and CB2R signaling^1,37,38^ has proven beneficial in reducing pain and inflammation. Treatment with cannabinoids such as CBD is associated with lower histological scores in inflammatory OA^32^ and reduced nociception in inflammatory arthritis.^40^ A recent observational study noted that 1 in 4 patients presenting with OA are using cannabinoid products for pain relief^8^ and a meta-analysis of medical cannabis use for OA found evidence of benefit for a subset of patients.^11^ Therefore, the ECS maybe an important pathway involved in perioperative pain.

The ECS is composed of multiple receptors, endogenous lipid ligands, and various enzymes that regulate endocannabinoid synthesis and degradation. Anandamide and 2-AG are the main lipid endocannabinoids, and while AEA mainly activates CB1R, 2-AG can activate CB1 and CB2 receptors (CB2R).^19,30^ Most CB1R are located on presynaptic neurons where they regulate and inhibit neurotransmitter release,^19,30^ whereas CB2R are predominately found in peripheral immune and hematopoietic cells where they mediate cytokine release and immune cell migration.^18^ Increases in circulating AEA concentrations have been shown to occur in patients with chronic pain syndromes, such as fibromyalgia^24^ and complex regional pain syndrome^23^ when compared with healthy individuals without pain. Patients with OA present with higher AEA and 2-AG synovial fluid levels as compared with healthy volunteers.^46^ Interestingly, a 2-week multimodal treatment with radon therapy in patients with knee OA revealed a significant reduction in plasma AEA levels and self-reported pain scores 6 months after treatment.^13^ Similarly, intrathecal morphine administration reduced peripheral AEA levels and postoperative pain in TKA patients.^2^ Regarding 2-AG, its central (CSF) and peripheral (synovial fluid) levels were significantly elevated and associated with higher postoperative opioid usage in patients who developed more severe postoperative pain after TKA^2,22^ further supporting the ECS as a therapeutic target for analgesia.

### 4.3. Endocannabinoid levels in diseased states

The relationship between endogenous endocannabinoid levels in healthy and diseased states in humans is not well-understood. A comparative study of endocannabinoid levels in patients with OA compared with healthy individuals demonstrated that synovial fluid PEA levels were significantly reduced while AEA and 2-AG levels were markedly higher.^46^ The authors reported that they were not able to detect 2-AG or AEA in synovial fluid of healthy individuals,^46^ similar to our own observation that these endocannabinoids were not detectable in many CSF samples from healthy individuals. Most of the studies that published CSF reference values for AEA stated undetectable to very low concentrations in healthy subjects similar to our results. Conversely, AEA levels are increased in patients with naïve schizophrenia,^16^ de novo Parkinson disease,^41^ or relapsing-remitting multiple sclerosis.^9^ We also observed increased CSF and peripheral blood levels of the N-acylethanolamines AEA, LEA, and OEA, which was consistent with a separate study of patients with OA or RA vs age-matched healthy subjects.^27^ Overall, in chronic disorders, especially those with a strong inflammatory component such as OA, altered levels of endocannabinoids are conceived as a maladaptive mechanism contributing to the disease or as an adaptive response aimed at restoring homeostasis.^3,10,23^ How these (mal)adaptive processes contribute to the (de)sensitization of pain receptors are still unknown, and it might involve different central and peripheral receptors and mechanisms.

### 4.4. Strengths and limitations

Our study has a number of strengths. The prospective observational cohort design in the setting of a standardized clinical protocol provides the opportunity to study endocannabinoid system markers in the context of the common clinical scenario of TKA for end-stage osteoarthritis. The sample collection occurred at well-defined time points relative to an established clinical protocol that is representative of typical orthopedic surgery and anesthetic practice. The use of a validated LC MS/MS method for measuring the analytes has a high accuracy and sensitivity with excellent test reproducibility. The observational crossover study design is beneficial for reducing bias by having each subject serve as their own control. We also assessed pain at multiple time points prospectively which reduces the risk of recall bias with retrospective data collection. Key limitations of the study include the lack of a randomized study design which would allow for causal inference and the reduction of potential bias with observational studies. The crossover study design did not provide a control group to measure the effect of the preoperative intake and anticipatory stress on endocannabinoids before surgery independent of acetaminophen exposure. Therefore, the differences between baseline measurements and the postacetaminophen time point may not be due to acetaminophen alone. The timing of the postoperative pain assessment was not standardized resulting in variability in when patients received their last analgesic medication and the pain assessment, which may have influenced the results. We did not specifically power the study to detect differences due to age or sex, but did adjust for these factors in the multivariable models. All patients received celecoxib and gabapentin as part of a standardized analgesic regimen, both of which have been shown to affect the EC system in animal models.^5,45^ The duration of outpatient follow-up was variable and likely nonrandom which affects the assessment of longer-term pain resolution. The study enrollment occurred at a tertiary referral center for orthopedic surgery and included a patient population that may not reflect some attributes of the general population or patients present for TKA which may limit the generalizability of our findings. The healthy control CSF samples analyzed in this study were from a commercial source so we do not have access to the individuals' health history, age, or OA status which limits interpretation of comparisons between the healthy controls and patients. The DVPRS was developed and validated in a military population which differed from our patient cohort.^42^

## 5. Conclusions

In summary, our data demonstrate that chronic inflammation, known to occur in patients with OA, is associated with higher CSF and plasma levels of AEA and its congeners. We also report an association between the CSF endocannabinoid levels and pain with 2-AG being associated with chronic preoperative pain and AEA being associated with acute postoperative pain. Overall, chronic pain, acetaminophen, and surgery have an impact on endogenous endocannabinoid CSF and blood levels. Modulation of the endocannabinoid system may be a beneficial therapeutic approach for supplementing existing multimodal analgesia strategies to manage postoperative pain. The results of this study support further study on the endocannabinoid system to identify patients at risk for postoperative pain and to identify novel approaches to individualizing postsurgery pain management strategies.

## Disclosures

The authors have no conflict of interest to declare.

## Supplemental digital content

Supplemental digital content associated with this article can be found online at http://links.lww.com/PR9/A360.
