# Supplementary material for: Endocannabinoids, perioperative pain, and acetaminophen in patients undergoing total knee arthroplasty: a prospective cohort study
Source: Pain Rep. 2026 Jan 30;11(2):e1369. doi: 10.1097/PR9.0000000000001369 (PMC12863799; doi:10.1097/PR9.0000000000001369)
Supplement: Supplementary file 1 [file painreports-11-e1369-s001.pdf]

STROBE Statement—checklist of items that should be included in reports of observational studies

	Item No	Recommendation	Page No
**Title and abstract**	1	(*a*) Indicate the study's design with a commonly used term in the title or the abstract	T
		(*b*) Provide in the abstract an informative and balanced summary of what was done and what was found	1
**Introduction**			
Background/rationale	2	Explain the scientific background and rationale for the investigation being reported	2
Objectives	3	State specific objectives, including any prespecified hypotheses	1-2
**Methods**			
Study design	4	Present key elements of study design early in the paper	2
Setting	5	Describe the setting, locations, and relevant dates, including periods of recruitment, exposure, follow-up, and data collection	3
Participants	6	(*a*) *Cohort study*—Give the eligibility criteria, and the sources and methods of selection of participants. Describe methods of follow-up<br>*Case-control study*—Give the eligibility criteria, and the sources and methods of case ascertainment and control selection. Give the rationale for the choice of cases and controls<br>*Cross-sectional study*—Give the eligibility criteria, and the sources and methods of selection of participants	3
		(*b*) *Cohort study*—For matched studies, give matching criteria and number of exposed and unexposed<br>*Case-control study*—For matched studies, give matching criteria and the number of controls per case	N/A
Variables	7	Clearly define all outcomes, exposures, predictors, potential confounders, and effect modifiers. Give diagnostic criteria, if applicable	3-4
Data sources/ measurement	8*	For each variable of interest, give sources of data and details of methods of assessment (measurement). Describe comparability of assessment methods if there is more than one group	4-5
Bias	9	Describe any efforts to address potential sources of bias	3,5,6
Study size	10	Explain how the study size was arrived at	5
Quantitative variables	11	Explain how quantitative variables were handled in the analyses. If applicable, describe which groupings were chosen and why	5-6
Statistical methods	12	(*a*) Describe all statistical methods, including those used to control for confounding	5-6
		(*b*) Describe any methods used to examine subgroups and interactions	6
		(*c*) Explain how missing data were addressed	5
		(*d*) *Cohort study*—If applicable, explain how loss to follow-up was addressed<br>*Case-control study*—If applicable, explain how matching of cases and controls was addressed<br>*Cross-sectional study*—If applicable, describe analytical methods taking account of sampling strategy	5
		(*e*) Describe any sensitivity analyses	N/A

Continued on next page

**Results**			
Participants	13*	(a) Report numbers of individuals at each stage of study—eg numbers potentially eligible, examined for eligibility, confirmed eligible, included in the study, completing follow-up, and analysed	3
		(b) Give reasons for non-participation at each stage	N/A
		(c) Consider use of a flow diagram	N/A
Descriptive data	14*	(a) Give characteristics of study participants (eg demographic, clinical, social) and information on exposures and potential confounders	Tbl 1
		(b) Indicate number of participants with missing data for each variable of interest	Tbl 2
		(c) *Cohort study*—Summarise follow-up time (eg, average and total amount)	
Outcome data	15*	*Cohort study*—Report numbers of outcome events or summary measures over time	Tbl 1
		Case-control study—Report numbers in each exposure category, or summary measures of exposure	
		Cross-sectional study—Report numbers of outcome events or summary measures	
Main results	16	(*a*) Give unadjusted estimates and, if applicable, confounder-adjusted estimates and their precision (eg, 95% confidence interval). Make clear which confounders were adjusted for and why they were included	6-9
		(*b*) Report category boundaries when continuous variables were categorized	6-9
		(*c*) If relevant, consider translating estimates of relative risk into absolute risk for a meaningful time period	
Other analyses	17	Report other analyses done—eg analyses of subgroups and interactions, and sensitivity analyses	
**Discussion**			
Key results	18	Summarise key results with reference to study objectives	9
Limitations	19	Discuss limitations of the study, taking into account sources of potential bias or imprecision. Discuss both direction and magnitude of any potential bias	12
Interpretation	20	Give a cautious overall interpretation of results considering objectives, limitations, multiplicity of analyses, results from similar studies, and other relevant evidence	10-12
Generalisability	21	Discuss the generalisability (external validity) of the study results	12
**Other information**			
Funding	22	Give the source of funding and the role of the funders for the present study and, if applicable, for the original study on which the present article is based	13

*Give information separately for cases and controls in case-control studies and, if applicable, for exposed and unexposed groups in cohort and cross-sectional studies.

Note: An Explanation and Elaboration article discusses each checklist item and gives methodological background and published examples of transparent reporting. The STROBE checklist is best used in conjunction with this article (freely available on the Web sites of PLoS Medicine at http://www.plosmedicine.org/, Annals of Internal Medicine at http://www.annals.org/, and Epidemiology at http://www.epidem.com/). Information on the STROBE Initiative is available at www.strobe-statement.org.

Supplementary Table 1. Complete study measurements and clinical data.

Baseline Measurements Plasma

Study ID	Sample Type	1_2LG (ng/ml)	2_AG (ng/ml)	AEA (ng/ml)	DEA (ng/ml)	LEA (pg/ml)	OEA LEA (pg/ml)	PEA LEA (pg/ml)	SEA LEA (pg/ml)
1	Plasma	2.32	0.7	0.66	75.44	2.31	4.22	4.5	3.49
2	Plasma	1.93	0.9	2.83	366.3	5.02	9.54	8.45	8.47
3	Plasma	1.08	1.3	1.29	230.42	2.63	4.97	5.4	6.42
4	Plasma	2.38	1.9	2.52	288.36	3.81	8.11	7.65	7.86
5	Plasma	2.24	1.8	2.04	261.07	4.12	7.23	7.25	7.19
6	Plasma	1.75	1.5	1.29	132.83	1.53	3.84	4.55	4.43
7	Plasma	2.67	2.6	1.5	180.5	3.38	8.44	5.15	5.86
8	Plasma	2.31	1.1	2.4	312.66	4.15	8.61	9.55	7.67
9	Plasma	3.33	4.5	0.8	114.13	1.21	3.82	3.1	3.25
10	Plasma	3.69	2.2	2.82	372.6	4.04	12.02	8.25	7.33
11	Plasma	2.84	1.4	1.67	161.03	2.92	4.84	4.8	4.83
12	Plasma	1.67	1.4	1.09	107.25	2.25	4.07	4.2	4.29
13	Plasma	7.72	1.4	4.17	484.19	8.88	19.77	25.95	19.19
14	Plasma	3.12	2.7	1.89	235.9	3.33	6.24	7	8.53
15	Plasma	3.96	4.3	1.51	174.81	2.17	4.99	4.95	6.83
16	Plasma	9.9	6.9	0.35	105.84	0.53	1.41	1.65	4.53
17	Plasma	10.38	4.1	4.75	516.72	5.62	17.42	15.9	14.01
18	Plasma	4.04	2.2	2.04	329.11	3.05	7.95	8.65	11.13
19	Plasma	2.5	1.6	1.71	339.66	2.64	5.01	7.2	7.05
20	Plasma	3.26	1.4	1.59	340.59	2.58	5.66	7.05	7.42
21	Plasma	4.09	2.4	1.02	172.52	1.07	3.39	4.1	3.59
22	Plasma	2.08	0.9	4.01	166.5	1.41	3.07	4.15	5.9
23	Plasma	7.23	2.3	2.55	353.6	5.71	11.09	12.15	11.41
24	Plasma	7.68	1.4	3.2	416.5	4.27	13.43	17	14.49
25	Plasma	2.25	1.6	2.45	392.88	4.09	10.7	12.25	10.09
26	Plasma	2.97	1.8	0.16	24.73	0.24	0.73	1.05	1.53
27	Plasma	3.51	1.6	1.5	249.24	3.82	6.24	6.45	6.77

28	Plasma	9.8	1.4	4.3	475.7	8.21	19.75	18.8	14.8
29	Plasma	5.03	1.6	1.56	212.75	2.27	6.16	7.55	6.33
30	Plasma	6.98	1.5	2.4	300	5.3	7.79	7.85	6.77
31	Plasma	5.64	0.6	2.74	360	5.22	12.7	12.65	10.59
32	Plasma	2.03	0.9	2.63	265	5.42	13.31	12.6	9.24
33	Plasma	3.23	1.4	5.42	735	7.57	17.25	15.4	9.78
34	Plasma	4.96	1.3	3.61	420	4.56	14.57	15.45	12.01
35	Plasma	12.41	7.1	2.84	450.43	4.21	11.04	12.8	6.53
36	Plasma	7.05	3	0.97	332.45	2.43	4.65	5.9	5.98
37	Plasma	2.45	0.9	2.99	386.67	5.03	10.82	9.85	6.76
38	Plasma	0.74	0.6	2.45	370	4.43	8.67	9.65	8.27
39	Plasma	9.12	1.9	1.6	159.09	2.88	6.43	6.55	6.06
40	Plasma	12	3.7	1.91	196.84	2.89	8.01	10.85	12.66
**Post IV Acetaminophen Measurements**									
1	Plasma	2.31	0.6	0.78	100	2.29	3.7	3.6	2.96
2	Plasma	1.39	0.8	1.33	200	2.83	5.65	4.9	5.16
3	Plasma	2.22	1.3	1.36	200	2.55	4.42	4.4	3.98
4	Plasma	2.69	2	2.57	280	3.99	10.08	10.25	7.87
5	Plasma	2.37	1.3	1.36	130	2.97	4.71	5.05	5.84
6	Plasma	1.41	1.4	1.33	140	1.53	4.59	5.1	5.36
7	Plasma	4.02	3.2	1.71	240	3.94	9.22	5.7	5.83
8	Plasma	1.37	1.9	1.6	220	2.68	6.52	6.25	4.65
9	Plasma	3.25	1.8	0.6	90	1.08	2.74	3.1	2.73
10	Plasma	3.38	2.5	2.73	370	3.69	11.45	8.15	6.71
11	Plasma	1.97	1.1	1.55	240	2.97	4.74	4.8	5.29
12	Plasma	12.31	4.5	2.16	340	4.06	11.45	13.05	11.65
13	Plasma	3.03	1.3	1.9	200	3.53	7.17	8.9	6.99
14	Plasma	3.9	3.4	1.74	270	2.44	5.5	5.65	4.76
15	Plasma	2.32	2.3	1.87	250	2.4	5.61	5.35	4.27
16	Plasma	9.31	3.1	1.77	190	3.02	7.2	11.7	10.6
17	Plasma	7.16	1.3	1.78	200	2.15	6.14	6.65	6.67
18	Plasma	5.38	1.8	0.98	190	1.43	4.24	6	5.24

19	Plasma	1.93	1.6	1.04	180	1.92	3.78	4.55	5.16
20	Plasma	4.05	3.4	1.01	190	1.6	3.4	5.6	4.66
21	Plasma	8.76	3.2	6.31	920	6.29	19.22	22.65	19.47
22	Plasma	6.18	1.9	5.01	700	7.21	19.4	25.95	21.46
23	Plasma	2.6	1.7	0.55	120	0.9	2.37	3.3	4.4
24	Plasma	0.4	0.5	0.11	10	0.18	0.58	5.05	8.79
25	Plasma	5.82	1.7	2.45	250	3.83	10.35	12.95	8.66
26	Plasma	4.64	1.5	3.5	370	6.8	12.32	14.9	11.69
27	Plasma	5.12	3.6	4.22	500	8.93	14.08	14.55	10.35
28	Plasma	10.24	1.4	3.02	240	5.68	12.25	10.3	9.69
29	Plasma	10.24	4.3	1.63	220	2.63	7.46	9.15	10.18
30	Plasma	5.8	2.2	5.83	780	11.6	25.08	24.65	26.75
31	Plasma	27.2	3.5	3.05	680	5.23	13.24	15.9	16.87
32	Plasma	10.69	1.5	4.7	400	10.66	23.55	23.5	21.49
33	Plasma	12.89	3.2	6.84	1050	8.92	22.37	23.7	19.93
34	Plasma	3.36	2	3.78	440	5.27	16.86	17.75	14.01
35	Plasma	6.68	1.8	4.15	640	10.14	14.73	16.2	13.54
36	Plasma	4.9	2.3	4.38	510	6.48	17.47	19.65	13.64
37	Plasma	16.43	3	4.11	460	8.98	19.59	17.9	17.36
38	Plasma	3.66	0.9	0.83	110	1.8	3.82	4.95	4.69
39	Plasma	12.86	2.3	4.15	280	6.24	15.86	16.2	13.25
40	Plasma	8.01	1.6	6.56	580	12.66	30.07	30.05	25.09

POD1 Measurements

1	Plasma	12.86	2.6	0.53	100	1.36	2.28	2.6	2.94
2	Plasma	6.36	2.5	0.79	100	1.62	3.3	3.6	3.63
3	Plasma	6.01	1.9	0.93	190	1.7	3.69	4.3	5.05
4	Plasma	6.46	3.2	0.49	130	0.74	1.92	2.65	3.89
5	Plasma	5.42	3.7	1.34	120	2.48	4.44	5.45	5.02
6	Plasma	3.98	3.7	0.53	90	0.86	2.8	3.55	2.75
7	Plasma	1.72	1.1	1.82	230	3.48	7	7.6	7.93
8	Plasma	3.16	1.2	1.86	190	3.34	6.46	6.2	5.55
9	Plasma	46.99	12.5	0.96	170	1.73	3.83	4.95	4.14

10	Plasma	24.6	12.7	0.64	150	1	2.96	2.75	3.91
11	Plasma	3.55	2.4	2	670	11.83	26	26.7	19.56
12	Plasma	4.3	2.1	0.88	180	1.72	3.41	4.3	4.15
13	Plasma	6.17	3	1.08	140	2.29	4.36	4.9	6.19
14	Plasma	2.11	1.8	1.29	210	1.85	3.96	4.1	4.36
15	Plasma	4.9	3.9	0.63	100	1.25	2.52	3.25	4.98
16	Plasma	6.22	5.9	0.91	230	1.17	3.6	4	6.4
17	Plasma	10.61	2.3	3	450	5.06	13.51	14.3	13.42
18	Plasma	1.76	2.3	1.01	180	1.19	3.85	4.45	5.41
19	Plasma	5.86	2.6	3.91	510	6.26	11.12	16.25	16.16
20	Plasma	3.83	2.7	0.55	280	0.92	2.55	3.8	5.87
21	Plasma	5.17	3.8	1.37	290	1.33	4.65	5.9	7.03
22	Plasma	2.33	1	0.91	160	2.85	4.73	6.05	6.85
23	Plasma	6.16	1.3	1.27	210	2.66	5.01	6.35	4.74
24	Plasma	13.75	1.8	1.16	220	1.47	5.19	7.15	7.17
25	Plasma	1.85	1	2.95	390	5.38	10.27	14.3	11.94
26	Plasma								
27	Plasma	8.11	3.1	2.79	400	5.78	10.72	10.3	11.45
28	Plasma	4.38	1.1	1.2	100	2.16	5.48	5.7	3.45
29	Plasma	5.65	1.9	1.3	210	1.89	5.43	7.8	7.13
30	Plasma	4.9	1.4	4.42	590	11.35	18.66	22.5	18.19
31	Plasma	15.77	3.8	4.52	780	8.65	16.16	18.45	16.52
32	Plasma	2.35	1.5	0.99	220	2.66	6.17	7	5.94
33	Plasma								
34	Plasma	6.23	0.9	0.55	100	1.12	4.45	6.25	5.82
35	Plasma	5	1.4	3.54	480	8.65	13.05	14.5	13.12
36	Plasma	1.9	1.5	2.37	280	3.34	10.1	11.7	9.53
37	Plasma	5.6	2.7	3.27	310	4.73	12.74	14.3	12.9
38	Plasma	3.86	1	1.3	450	4.4	10.98	16.05	15.72
39	Plasma	8.43	3	3.37	290	4.66	12.23	12.9	11.83
40	Plasma	11.6	3.2	1.63	140	3.67	9.54	9.55	7.39

Study ID	Sample Type	2AG (pg/ml)	2AGE (pg/ml)	AEA (pg/ml)	DEA (pg/ml)	DH-g-LEA (pg/ml)	LEA (pg/ml)	NADA (pg/ml)	O-AEA (pg/ml)	ODA (pg/ml)	OEA (pg/ml)	OLA (pg/ml)	PEA (pg/ml)	SEA (pg/ml)
1	CSF	15.29	16.82	11.75	5.2	4.95	13.93	118.52	8.38	62.723	27.93	0.747	54.63	1.149
2	CSF	13.07	75.86	7.47	3.97	2.88	17.4	70.24	8.21	30.401	33.84	1.821	44.05	1.113
3	CSF	11.8	213.69	9.69	5.22	2.09	12.31	81.96	7.13	15.726	32.82	0.758	48.85	1.812
4	CSF	9.87	49.85	9.83	6.04	2.4	20.65	62.01	10.58	19.216	53.81	0.822	43.93	1.766
5	CSF	18.26	80.86	5.43		1.86	12.95	79.43	5.73	18.51	30.59	1.501	51.35	1.991
6	CSF	18.08	198.27	8.32	2.01	1.09	12.67	67.91	8.96	9.997	30.75	3.306	36.76	0.927
7	CSF	8.36	39.79	9.74	3.54	0.98	10.45	51.45	11.88	6.974	37.76	32.65	46.06	1.405
8	CSF	14.13	108.05	11.08	6.67	1.8	10.7	70.83	8.94	10.179	41.45	31.891	41.91	1.541
9	CSF	15.35	36.49	7		1.01	13.11	63.05	4.01	3.647	31.25	40.08	65.29	2.166
10	CSF	22.86	25.77	11.88	7.01	1.9	13.19	71.76	8.73	3.113	44.82	78.06	47.78	1.655
11	CSF	10.89	100.01	12.48	5.25	1.28	16.34	53.41	13.86	7.846	41.22	1.165	48.53	2.494
12	CSF	9.49	164.58	10.65		0.83	14.22	63.77	16.43	5.783	29.97	1.18	36.69	2.017
13	CSF	15.12	136.36	5.02		1.04	12.12	65.69	14.89	4.076	37.35	0.953	38.95	1.94
14	CSF	22.66	14.59	6.39	1.89	1.65	14.95	69.85	8.52	4.514	47.64	1.783	42.56	2.103
15	CSF	13.85	110.92	10.86	2.37	0.83	14.49	49.86	7.87	5.803	33.92	0.967	42.85	2.007
16	CSF	35.86	108.09	13.25	19.92	1.65	18.02	60.97	22.82	3.873	159.69	0.807	50.6	1.872
17	CSF	9.99	107.75	13.44	4.6	1.08	8.57	71.58	8.49	2.807	47.15	0.971	43.58	2.269
18	N/A													
19	CSF	14.12	23.29	5.74	3.38	1.11	14.81	80.18	12	3.896	41.67	1.098	40.52	1.91
20	CSF	16.07	32.13	4.13	5.36	1.06	14.87	55.35	8.03	4.222	32.42	0.934	41.36	1.805
21	CSF	20.47	132.92	10.81	5.19	1.16	13.31	64.27	3.91	1.868	41.62	1.838	40.98	2.1
22	CSF	11.48	28.08	15.08	2.76	0.55	13.45	61.19	7.01	3.681	26.25	1.033	42.31	1.741
23	CSF	8.79	38.31	9.53		0.83	14.67	51.72	7.44	2.529	28.87	1.141	41.42	1.973
24	CSF	19.89	112.64	18.98		0.94	18.18	68.2	9.32	6.282	43.44	1.467	46.58	1.843
25	CSF	15.47	30.76	12.2	2.71	0.46	10.03	77.36	9.07	1.954	28.64	1.077	38.81	3.719
26	CSF	19.68	114.21	7.16		1.03	24.56	66.71	6.17	1.724	53.84	2.848	55.14	1.939
27	CSF	25.61	32.27	10.87	8.35	1.11	13.87	58.37	6.97	1.373	37.25	1.979	39.15	1.596
28	CSF	12.86	21.54	13.68	4.89	1.25	13.56	75.12	9.12	3.601	40.33	1.869	39.93	2.202
29	CSF	14.76	20.21	10.81	2.71	1.11	11.44	57.36	6.88	1.536	23.43	1.518	35.36	1.466

30	CSF	14.5	104.6	10.53	7.07	0.68	16.87	57.19	12.91	2.513	56.03	82.408	42.87	3.055
31	CSF	50.47	13.46	8.62	6.93	0.75	17.34	60.07	7.36	2.199	56.48	40.788	43.17	1.816
32	CSF	20.35	9.32	9.81	3.23	1.33	13.9	60.99	8.29	2.262	43.84	81.566	43.69	2.279
33	CSF	13.74	57.66	13.43	4.42	1.34	11.85	58.17	6.42	3.608	53.35	3.39	51.72	2.198
34	CSF	11.51	57.12	8.37	3.26	0.67	16.05	61.74	13.08	3.397	51.93	2.306	40.89	2.164
35	CSF	7.45	71.04	8.56	4.26	0.36	13.58	67.54	6.25	11.76	30.57	1.218	38.31	2.156
36	CSF	12.66	65.18	6.19	3.87	0.62	13.17	62.04	9.5	1.489	28.59	1.277	34.5	2.352
37	CSF	47.84	84.79	9.4	5.53	0.78	16.43	60.21	12.14	1.679	34.94	12.202	41.08	1.774
38	CSF	30.22	17.96	4.93	3.56	1.02	14.6	49.48	6.79	4.394	28.08	1.296	41.07	2.193
39	CSF	20.78	16.17	10.5	1.9	0.85	14	52.54	8.01	1.963	36.15	1.329	40.21	2.197
40	CSF	21.18	58.93	3.7	1.73	0.62	13.53	50.22	9.26	0.955	40.96	0.938	40.51	1.64

Clinical data

ID	Timepoint	dvprs_rest	dvprs_movement	Max_pain_binary	pain_rest_binary	pain_movement_binary	pain_activity_binary	pain_sleep_binary	pain_mood_binary	pain_stress_binary	pain_often_binary	pain_time_length_binary	pain_resolved_binary	Max pain_again	pain_movement_again	pain_activity_again	max pain_4cat	pain_rest_4cat	pain_movement_4cat	pain_activity_4cat	pain_sleep_4cat	pain_mood_4cat	pain_stress_4cat
1	1	1	2	2	2	1	2	1	1	1	1	2	0	2	2	2	3	1	3	3	1	0	0
2	1	1	2	2	1	1	2	1	2	2	1	2	1	1	1	1	2	1	2	2	0	2	2
3	1	2	2	2	2	1	1	2	1	1	2	1	0	1	1	1	2	2	2	0	1	0	0
4	1	1	1	1	2	0	1	2	1	2	1	2	0	1	1	1	2	1	2	2	2	0	2
5	1	1	2	2	1	1	1	1	1	1	2	2	1	2	2	1	2	1	2	2	0	0	0
6	1			2	2	1	2	2	1	1	1	2	1	2	1	2	2	1	2	2	2	0	0
7	1			1	1	0	1	1	2	2	2	2	0	1	1	1	1	0	1	0	1	1	2
8	1	1	1	1	2	1	1	1	2	2	1	2	0	1	1	1	1	1	1	1	1	1	1

9	1	1	2	2	2	1	1	1	2	2	1	2	1	1	1	1	2	2	2	1	1	1	1
10	1	1	2	2	2	1	2	1	2	2	1	2	1	1	1	1	2	1	2	2	0	2	1
11	1	1	2	2	1	1	2	2	2	2	2	2	0	1	1	1	2	1	2	2	2	2	3
12	1	1	2	1	2	0	1	2	2	2	2	1	1	1	1	1	1	1	1	1	1	1	1
13	1	1	1	2	2	1	2	1	1	1	1	2	1	2	1	2	3	1	2	3	0	0	0
14	1	1	2	2	2	1	2	2	2	2	1	2	0	2	2	2	3	1	2	3	3	2	2
15	1	1	2	2	2	1	2	2	2	2	1	2	0	2	2	2	2	2	2	2	2	1	2
16	1	1	2	2	2	1	2	1	2	2	2	2	1	2	2	2	3	1	3	2	1	1	3
17	1			1	1	0	1	1	2	2	2	2	0	1	1	1	1	0	1	1	1	1	1
18	1	1	1																				
19	1	1	2	2	1	1	2	2	1	1	2	2	0	2	2	2	3	1	2	3	2	0	0
20	1	1	2	2	2	1	2	2	2	2	2	2	0	2	2	2	3	1	3	3	3	2	2
21	1	1	2	2	2	1	2	2	1	2	2	1	1	2	2	2	2	1	2	2	2	0	1
22	1	1	1	2	2	0	2	1	1	1	2	2	1	1	1	1	2	1	1	2	0	0	0
23	1			1	1	0	1	2	1	1	2	2	1	1	1	1	1	0	1	1	1	0	0
24	1	2	1	2	2	0	2	2	2	2	2	2	1	2	1	2	2	2	2	2	2	2	3

25	1			1	1	0	1	1	1	1	2	2	0	1	1	1	1	0	1	1	1	0	0
26	1	1	2	2	1	1	2	2	2	2	2	2	0	2	1	2	3	0	2	3	1	2	1
27	1	1	2	2	2	1	2	2	1	1	1	2	0	2	2	1	2	1	2	2	2	1	0
28	1	1	1	1	1	0	1	1	2	2	2	2	1	1	1	1	2	0	1	2	1	1	1
29	1	2	2	2	2	1	2	2	2	2	1	2	1	2	2	2	3	2	3	3	3	2	2
30	1			2	2	1	1	2	2	1	2	2	1	2	2	1	3	3	3	2	2	1	0
31	1	1	2	2	1	1	2	2	1	2	1	1	1	2	2	2	3	0	3	3	3	1	2
32	1			2	1	1	2	2	2	2	1	2	1	2	2	2	3	0	3	3	3	3	3
33	1			1	1	0	1	1	1	1	2	2	1	1	1	1	1	0	1	1	1	0	0
34	1	1	1	1	1	0	1	2	2	2	2	2	1	1	1	1	1	0	1	1	3	2	1
35	1	1	1	1	1	0	1	2	1	2	2	2	1	1	1	1	2	0	1	2	2	0	2
36	1	1	2	2	1	1	2	1	1	1	2	2	0	2	2	2	3	0	3	3	0	0	0
37	1	1	2	2	2	1	2	2	2	2	1	1	1	2	2	2	3	2	3	3	3	2	2
38	1	2	2	2	2	1	1	2	2	2	1	2	0	2	2	1	3	2	3	2	2	2	2
39	1	1	2	2	1	1	2	1	2	2	2	1	0	2	2	2	3	0	2	3	0	2	2

40	1			2	1	1	2	1	2	2	2	2	1	2	2	2	3	0	3	3	0	2	2
1	2			2	2	1	2	1	1	1	1	2	0	2	2	2	3	1	3	3	1	0	0
2	2			2	1	1	2	1	2	2	1	2	1	1	1	1	2	1	2	2	0	2	2
3	2			2	2	1	1	2	1	1	2	1	0	1	1	1	2	2	2	0	1	0	0
4	2			1	2	0	1	2	1	2	1	2	0	1	1	1	2	1	2	2	2	0	2
5	2			2	1	1	1	1	1	1	2	2	1	2	2	1	2	1	2	2	0	0	0
6	2	1	1	2	2	1	2	2	1	1	1	2	1	2	1	2	2	1	2	2	2	0	0
7	2	1	1	1	1	0	1	1	2	2	2	2	0	1	1	1	1	0	1	0	1	1	2
8	2			1	2	1	1	1	2	2	1	2	0	1	1	1	1	1	1	1	1	1	1
9	2			2	2	1	1	1	2	2	1	2	1	1	1	1	2	2	2	1	1	1	1
10	2			2	2	1	2	1	2	2	1	2	1	1	1	1	2	1	2	2	0	2	1
11	2			2	1	1	2	2	2	2	2	2	0	1	1	1	2	1	2	2	2	2	3
12	2			1	2	0	1	2	2	2	2	1	1	1	1	1	1	1	1	1	1	1	1
13	2			2	2	1	2	1	1	1	1	2	1	2	1	2	3	1	2	3	0	0	0
14	2			2	2	1	2	2	2	2	1	2	0	2	2	2	3	1	2	3	3	2	2
15	2			2	2	1	2	2	2	2	1	2	0	2	2	2	2	2	2	2	2	1	2
16	2			2	2	1	2	1	2	2	2	2	1	2	2	2	3	1	3	2	1	1	3
17	2	1	1	1	1	0	1	1	2	2	2	2	0	1	1	1	1	0	1	1	1	1	1
18	2																						
19	2			2	1	1	2	2	1	1	2	2	0	2	2	2	3	1	2	3	2	0	0

20	2			2	2	1	2	2	2	2	2	2	0	2	2	2	3	1	3	3	3	2	2
21	2			2	2	1	2	2	1	2	2	1	1	2	2	2	2	1	2	2	2	0	1
22	2			2	2	0	2	1	1	1	2	2	1	1	1	1	2	1	1	2	0	0	0
23	2	1	1	1	1	0	1	2	1	1	2	2	1	1	1	1	1	0	1	1	1	0	0
24	2			2	2	0	2	2	2	2	2	2	1	2	1	2	2	2	2	2	2	2	3
25	2	1	1	1	1	0	1	1	1	1	2	2	0	1	1	1	1	0	1	1	1	0	0
26	2			2	1	1	2	2	2	2	2	2	0	2	1	2	3	0	2	3	1	2	1
27	2			2	2	1	2	2	1	1	1	2	0	2	2	1	2	1	2	2	2	1	0
28	2			1	1	0	1	1	2	2	2	2	1	1	1	1	2	0	1	2	1	1	1
29	2			2	2	1	2	2	2	2	1	2	1	2	2	2	3	2	3	3	3	2	2
30	2	1	1	2	2	1	1	2	2	1	2	2	1	2	2	1	3	3	3	2	2	1	0
31	2			2	1	1	2	2	1	2	1	1	1	2	2	2	3	0	3	3	3	1	2
32	2	1	1	2	1	1	2	2	2	2	1	2	1	2	2	2	3	0	3	3	3	3	3
33	2	1	1	1	1	0	1	1	1	1	2	2	1	1	1	1	1	0	1	1	1	0	0
34	2			1	1	0	1	2	2	2	2	2	1	1	1	1	1	0	1	1	3	2	1

35	2			1	1	0	1	2	1	2	2	2	1	1	1	1	2	0	1	2	2	0	2
36	2			2	1	1	2	1	1	1	2	2	0	2	2	2	3	0	3	3	0	0	0
37	2			2	2	1	2	2	2	2	1	1	1	2	2	2	3	2	3	3	3	2	2
38	2			2	2	1	1	2	2	2	1	2	0	2	2	1	3	2	3	2	2	2	2
39	2			2	1	1	2	1	2	2	2	1	0	2	2	2	3	0	2	3	0	2	2
40	2	1	2	2	1	1	2	1	2	2	2	2	1	2	2	2	3	0	3	3	0	2	2
1	3	2	2	2	2	1	2	1	1	1	1	2	0	2	2	2	3	1	3	3	1	0	0
2	3	1	1	2	1	1	2	1	2	2	1	2	1	1	1	1	2	1	2	2	0	2	2
3	3	1	1	2	2	1	1	2	1	1	2	1	0	1	1	1	2	2	2	0	1	0	0
4	3	1	1	1	2	0	1	2	1	2	1	2	0	1	1	1	2	1	2	2	2	0	2
5	3	1	1	2	1	1	1	1	1	1	2	2	1	2	2	1	2	1	2	2	0	0	0
6	3	1	1	2	2	1	2	2	1	1	1	2	1	2	1	2	2	1	2	2	2	0	0
7	3	2	2	1	1	0	1	1	2	2	2	2	0	1	1	1	1	0	1	0	1	1	2
8	3	2	2	1	2	1	1	1	2	2	1	2	0	1	1	1	1	1	1	1	1	1	1
9	3	1	1	2	2	1	1	1	2	2	1	2	1	1	1	1	2	2	2	1	1	1	1
10	3	1	1	2	2	1	2	1	2	2	1	2	1	1	1	1	2	1	2	2	0	2	1
11	3	1	2	2	1	1	2	2	2	2	2	2	0	1	1	1	2	1	2	2	2	2	3
12	3	1	2	1	2	0	1	2	2	2	2	1	1	1	1	1	1	1	1	1	1	1	1
13	3	1	1	2	2	1	2	1	1	1	1	2	1	2	1	2	3	1	2	3	0	0	0
14	3	1	2	2	2	1	2	2	2	2	1	2	0	2	2	2	3	1	2	3	3	2	2

15	3	1	2	2	2	1	2	2	2	2	1	2	0	2	2	2	2	2	2	2	2	1	2
16	3	2	2	2	2	1	2	1	2	2	2	2	1	2	2	2	3	1	3	2	1	1	3
17	3	1	1	1	1	0	1	1	2	2	2	2	0	1	1	1	1	0	1	1	1	1	1
18	3	1	1																				
19	3	1	1	2	1	1	2	2	1	1	2	2	0	2	2	2	3	1	2	3	2	0	0
20	3	1	2	2	2	1	2	2	2	2	2	2	0	2	2	2	3	1	3	3	3	2	2
21	3	1	1	2	2	1	2	2	1	2	2	1	1	2	2	2	2	1	2	2	2	0	1
22	3	1	1	2	2	0	2	1	1	1	2	2	1	1	1	1	2	1	1	2	0	0	0
23	3	1	2	1	1	0	1	2	1	1	2	2	1	1	1	1	1	0	1	1	1	0	0
24	3	1	1	2	2	0	2	2	2	2	2	2	1	2	1	2	2	2	2	2	2	2	3
25	3	1	1	1	1	0	1	1	1	1	2	2	0	1	1	1	1	0	1	1	1	0	0
26	3	1	1	2	1	1	2	2	2	2	2	2	0	2	1	2	3	0	2	3	1	2	1
27	3	1	1	2	2	1	2	2	1	1	1	2	0	2	2	1	2	1	2	2	2	1	0
28	3	1	1	1	1	0	1	1	2	2	2	2	1	1	1	1	2	0	1	2	1	1	1
29	3	1	1	2	2	1	2	2	2	2	1	2	1	2	2	2	3	2	3	3	3	2	2

30	3	1	1	2	2	1	1	2	2	1	2	2	1	2	2	1	3	3	3	2	2	1	0
31	3	1	1	2	1	1	2	2	1	2	1	1	1	2	2	2	3	0	3	3	3	1	2
32	3	1	2	2	1	1	2	2	2	2	1	2	1	2	2	2	3	0	3	3	3	3	3
33	3	1	1	1	1	0	1	1	1	1	2	2	1	1	1	1	1	0	1	1	1	0	0
34	3	1	1	1	1	0	1	2	2	2	2	2	1	1	1	1	1	0	1	1	3	2	1
35	3	1	1	1	1	0	1	2	1	2	2	2	1	1	1	1	2	0	1	2	2	0	2
36	3	1	2	2	1	1	2	1	1	1	2	2	0	2	2	2	3	0	3	3	0	0	0
37	3	1	2	2	2	1	2	2	2	2	1	1	1	2	2	2	3	2	3	3	3	2	2
38	3	1	1	2	2	1	1	2	2	2	1	2	0	2	2	1	3	2	3	2	2	2	2
39	3	1	1	2	1	1	2	1	2	2	2	1	0	2	2	2	3	0	2	3	0	2	2
40	3	1	2	2	1	1	2	1	2	2	2	2	1	2	2	2	3	0	3	3	0	2	2

Supplementary Table 2. Exploratory correlations analysis between study variables.

Baseline Correlations

**Variable**	**by Variable**	**Spearman ρ**	**Prob>|ρ|**	**FDR Prob>|ρ|**
DEA	AEA	0.8266	<0.0001	0.0406
LEA	AEA	0.8207	<.0001	0.0406
LEA	DEA	0.8233	<.0001	0.0406
OEA	AEA	0.8489	<.0001	0.0406
OEA	DEA	0.8686	<.0001	0.0406
OEA	LEA	0.9302	<.0001	0.0406
PEA	AEA	0.8479	<.0001	0.0406
PEA	DEA	0.8949	<.0001	0.0406
PEA	LEA	0.8891	<.0001	0.0406
PEA	OEA	0.9541	<.0001	0.0406
pain_activity	dvprs_movement	0.7304	<.0001	0.0406
pain_stress	pain_mood	0.6916	<.0001	0.0406
SEA	AEA	0.7641	<.0001	0.0406
SEA	DEA	0.7856	<.0001	0.0406
SEA	LEA	0.776	<.0001	0.0406
SEA	OEA	0.8443	<.0001	0.0406
SEA	PEA	0.8925	<.0001	0.0406
2_AG	1_2LG	0.5327	0.0004	0.0812
2_AG_CSF	dvprs_movement	0.4958	0.0013	0.175933333
PEA_CSF	DH-g-LEA_CSF	0.4874	0.0016	0.1624
ODA_CSF	DH-g-LEA_CSF	0.4574	0.0034	0.27608
ODA_CSF	1_2LG	-0.4291	0.0064	0.433066667
OEA_CSF	age	0.4215	0.0075	0.435
NADA_CSF	DH-g-LEA_CSF	0.4106	0.0094	0.47705
2_AG_CSF	pain_activity	0.3993	0.0118	0.532311111
pain_stress	pain_sleep	0.3861	0.0139	0.56434
PEA_CSF	OEA_CSF	0.3901	0.0141	0.520418182
ODA_CSF	2_AG_CSF	-0.3864	0.0151	0.510883333
OEA_CSF	LEA_CSF	0.386	0.0152	0.474707692
SEA	age	0.3789	0.0159	0.4611
AEA_CSF	pain_sleep	-0.3831	0.0161	0.435773333
2_AG_CSF	pain_mood	0.3811	0.0167	0.4237625
PEA_CSF	DEA_CSF	0.4177	0.0174	0.415552941
AEA_CSF	dvprs_movement	-0.3663	0.0218	0.491711111
2_AGE_CSF	pain_activity	-0.3642	0.0226	0.482926316
pain_sleep	2_AG	-0.3568	0.0238	0.48314
SEA	Male sex	-0.3561	0.0241	0.465933333
PEA_CSF	ODA_CSF	0.3494	0.0292	0.538872727
OEA_CSF	DEA_CSF	0.3814	0.0312	0.550747826
dvprs_movement	dvprs_rest	0.3408	0.0314	0.531183333
ODA_CSF	dvprs_rest	0.3447	0.0316	0.513184

age	1_2LG	0.3376	0.0331	0.516869231
PEA	1_2LG	0.3355	0.0343	0.51577037
ODA_CSF	NADA_CSF	0.3399	0.0343	0.49735
DH-g-LEA_CSF	1_2LG	-0.3382	0.0352	0.4928
pain_activity	Male sex	-0.3332	0.0356	0.481786667
dvprs_rest	2_AG	-0.3327	0.0359	0.470174194
pain_sleep	dvprs_movement	0.3304	0.0373	0.47324375
bmi	AEA	-0.329	0.0382	0.469975758
2_AG_CSF	pain_stress	0.3282	0.0414	0.494364706
LEA	2_AG	-0.3209	0.0435	0.5046
dvprs_rest	1_2LG	-0.32	0.0441	0.49735
2_AGE_CSF	dvprs_movement	-0.3203	0.0468	0.513535135
AEA	2_AG	-0.3159	0.047	0.502157895
DH-g-LEA_CSF	DEA_CSF	0.3483	0.0508	0.528841026
O-AEA_CSF	age	0.3074	0.057	0.57855
NADA_CSF	dvprs_movement	-0.306	0.0581	0.575331707
SEA	1_2LG	0.3002	0.0598	0.578066667
OEA_CSF	1_2LG	0.3014	0.0622	0.587283721
SEA_CSF	DH-g-LEA_CSF	-0.3011	0.0625	0.576704545
2_AG_CSF	pain_sleep	0.3008	0.0628	0.566595556
pain_sleep	dvprs_rest	0.2935	0.066	0.582521739
pain_stress	OEA	0.2899	0.0696	0.601225532
DH-g-LEA_CSF	dvprs_rest	0.2921	0.0711	0.6013875
pain_sleep	DEA	0.2878	0.0717	0.594085714
ODA_CSF	pain_mood	-0.2916	0.0717	0.582204
AEA_CSF	pain_activity	-0.2911	0.0722	0.574768627
SEA	pain_stress	0.2861	0.0735	0.573865385
OEA	1_2LG	0.282	0.0779	0.596743396
SEA_CSF	Male sex	-0.2851	0.0785	0.590203704
ODA_CSF	bmi	-0.2836	0.0802	0.592021818
ODA_CSF	pain_activity	-0.2813	0.0828	0.6003
PEA	Male sex	-0.2751	0.0858	0.611136842
OEA_CSF	O-AEA_CSF	0.2769	0.0879	0.6153
LEA_CSF	dvprs_movement	0.2748	0.0905	0.622762712
pain_sleep	PEA	0.2696	0.0925	0.625916667
age	AEA	0.2683	0.0941	0.626304918
pain_stress	dvprs_movement	0.2667	0.0962	0.629954839
pain_stress	PEA	0.2657	0.0975	0.628333333
DEA_CSF	AEA_CSF	0.2973	0.0985	0.624859375
SEA_CSF	LEA	0.2684	0.0985	0.615246154
pain_sleep	LEA	0.2635	0.1004	0.617612121
DEA	1_2LG	0.2625	0.1018	0.616877612
ODA_CSF	dvprs_movement	-0.2637	0.1048	0.625717647
OEA_CSF	SEA	0.2636	0.105	0.617826087
SEA_CSF	ODA_CSF	-0.2621	0.1069	0.62002
pain_mood	dvprs_movement	0.2585	0.1072	0.613002817
dvprs_movement	Male sex	-0.2568	0.1097	0.618586111

LEA_CSF	DEA_CSF	0.2869	0.1114	0.619567123
bmi	age	-0.2538	0.1141	0.626008108
pain_stress	pain_activity	0.2537	0.1142	0.618202667
pain_sleep	OEA	0.2536	0.1144	0.611136842
O-AEA_CSF	pain_activity	-0.2554	0.1166	0.6148
pain_sleep	pain_activity	0.252	0.1168	0.607958974
SEA_CSF	DEA	0.2534	0.1195	0.614139241
OEA_CSF	OEA	0.2503	0.1243	0.6308225
SEA	pain_mood	0.2461	0.1258	0.630553086
pain_sleep	age	-0.2459	0.1261	0.62434878
Male sex	OEA	-0.2428	0.1312	0.641773494
pain_mood	OEA	0.2423	0.132	0.638
pain_mood	1_2LG	0.2422	0.1322	0.631449412
2_AG_CSF	Male sex	-0.2416	0.1383	0.652904651
PEA	age	0.2381	0.1391	0.649133333
pain_mood	bmi	0.2351	0.1442	0.665286364
pain_mood	PEA	0.2338	0.1465	0.668303371
SEA_CSF	1_2LG	0.236	0.148	0.667644444
pain_activity	AEA	-0.2327	0.1485	0.662538462
OEA_CSF	LEA	0.2338	0.152	0.670782609
SEA_CSF	dvprs_rest	-0.2323	0.1547	0.675356989
pain_mood	Male sex	-0.2278	0.1574	0.679834043
PEA	2_AG	-0.2266	0.1597	0.682507368
ODA_CSF	2_AGE_CSF	0.2291	0.1605	0.67878125
pain_sleep	bmi	0.2251	0.1626	0.680573196
2_AGE_CSF	2_AG_CSF	-0.2279	0.1628	0.674457143
OEA_CSF	pain_mood	0.2254	0.1677	0.687739394
2_AGE_CSF	dvprs_rest	0.225	0.1684	0.683704
pain_activity	LEA	-0.2217	0.1691	0.679748515
SEA_CSF	PEA	0.2229	0.1726	0.687015686
Male sex	LEA	-0.2196	0.1733	0.683104854
OEA_CSF	2_AG_CSF	0.2221	0.1742	0.68005
O-AEA_CSF	LEA_CSF	0.2217	0.175	0.676666667
PEA_CSF	LEA_CSF	0.2213	0.1759	0.673730189
NADA_CSF	1_2LG	-0.22	0.1783	0.676540187
pain_stress	bmi	0.2162	0.1802	0.677418519
OEA	2_AG	-0.2151	0.1825	0.679770642
NADA_CSF	LEA_CSF	-0.2148	0.1892	0.69832
PEA_CSF	PEA	-0.2146	0.1896	0.693491892
OEA_CSF	DH-g-LEA_CSF	0.2144	0.19	0.68875
pain_mood	pain_sleep	0.2115	0.1901	0.683014159
2_AG_CSF	bmi	0.2142	0.1903	0.677735088
LEA_CSF	2_AG_CSF	0.2121	0.1948	0.687728696
pain_activity	OEA	-0.2083	0.1971	0.68985
2_AGE_CSF	OEA	-0.2106	0.198	0.687076923
NADA_CSF	bmi	-0.2099	0.1997	0.68710339
DEA_CSF	2_AGE_CSF	0.2319	0.2016	0.687811765

dvprs_rest	age	-0.2059	0.2025	0.685125
age	OEA	0.2057	0.203	0.681140496
DEA_CSF	pain_stress	-0.2303	0.2048	0.681547541
pain_stress	1_2LG	0.2027	0.2097	0.692180488
2_AGE_CSF	SEA	-0.2049	0.2109	0.690527419
age	DEA	0.2014	0.2127	0.6908496
OEA_CSF	dvprs_rest	-0.2016	0.2184	0.703733333
O-AEA_CSF	2_AGE_CSF	0.2012	0.2193	0.701069291
PEA	bmi	-0.1981	0.2205	0.699398438
pain_mood	pain_activity	0.1973	0.2222	0.699327132
SEA	bmi	-0.1968	0.2236	0.69832
PEA_CSF	DEA	-0.199	0.2246	0.69608855
NADA_CSF	pain_mood	-0.1987	0.2253	0.692968182
dvprs_movement	2_AG	-0.1937	0.2311	0.705463158
pain_activity	DEA	-0.1935	0.2314	0.701107463
dvprs_movement	AEA	-0.1927	0.2336	0.70253037
AEA_CSF	AEA	0.1949	0.2345	0.700051471
SEA_CSF	OEA	0.193	0.2391	0.708573723
PEA_CSF	O-AEA_CSF	-0.1903	0.2459	0.723444928
bmi	OEA	-0.1862	0.25	0.730215827
LEA_CSF	pain_activity	0.1884	0.2506	0.72674
OEA_CSF	PEA	0.1885	0.2506	0.721585816
DEA_CSF	LEA	0.2086	0.2519	0.720221127
OEA_CSF	AEA	0.1868	0.2549	0.723702098
PEA_CSF	1_2LG	-0.186	0.2568	0.724033333
PEA_CSF	AEA_CSF	0.1854	0.2584	0.72352
Male sex	DEA	-0.1826	0.2593	0.721067123
DEA	2_AG	-0.1817	0.2619	0.723342857
Male sex	2_AG	0.181	0.2636	0.723118919
2_AGE_CSF	PEA	-0.1832	0.2643	0.720173154
LEA	1_2LG	0.1786	0.2702	0.731341333
PEA_CSF	Male sex	-0.1788	0.2761	0.742361589
bmi	LEA	-0.1754	0.279	0.745223684
pain_stress	Male sex	-0.1754	0.2791	0.740618301
AEA	1_2LG	0.1751	0.2799	0.737918182
2_AGE_CSF	age	0.1766	0.2822	0.739181935
SEA_CSF	AEA	0.1765	0.2826	0.735484615
2_AGE_CSF	LEA	-0.1751	0.2863	0.740368153
SEA_CSF	SEA	0.1726	0.2934	0.753926582
ODA_CSF	2_AG	-0.172	0.2952	0.753781132
2_AGE_CSF	AEA	-0.1715	0.2965	0.75236875
2_AGE_CSF	pain_sleep	-0.1715	0.2965	0.747695652
SEA_CSF	2_AG	0.1702	0.3002	0.752353086
NADA_CSF	pain_stress	-0.1683	0.3058	0.76168589
LEA_CSF	2_AG	-0.168	0.3066	0.759021951
pain_stress	DEA	0.1657	0.3069	0.75516
SEA	pain_sleep	0.1653	0.308	0.753301205

O-AEA_CSF	SEA	0.1659	0.3128	0.76045988
PEA_CSF	pain_sleep	-0.1647	0.3164	0.764633333
2_AGE_CSF	2_AG	0.1629	0.3217	0.77284142
pain_mood	dvprs_rest	0.1607	0.3218	0.768534118
AEA_CSF	2_AG_CSF	-0.1623	0.3237	0.768550877
LEA_CSF	pain_sleep	0.1589	0.3341	0.788631395
bmi	DEA	-0.1561	0.3363	0.789235838
O-AEA_CSF	pain_mood	0.1577	0.3378	0.7882
2_AG_CSF	AEA	-0.1573	0.3388	0.786016
DH-g-LEA_CSF	PEA	-0.1548	0.3466	0.799543182
DEA_CSF	2_AG	-0.1719	0.3468	0.795484746
O-AEA_CSF	LEA	0.1524	0.3542	0.807894382
SEA	2_AG	-0.1498	0.3561	0.807690503
pain_sleep	Male sex	0.1471	0.3651	0.823503333
O-AEA_CSF	2_AG_CSF	-0.149	0.3653	0.81940221
NADA_CSF	pain_activity	-0.1489	0.3658	0.816015385
O-AEA_CSF	OEA	0.1472	0.3713	0.82375847
DH-g-LEA_CSF	2_AG	-0.147	0.3717	0.82016413
OEA_CSF	AEA_CSF	0.1469	0.3723	0.817047568
age	2_AG	0.1443	0.3743	0.81702043
NADA_CSF	DEA	0.1455	0.3766	0.81764492
pain_activity	1_2LG	0.142	0.382	0.824957447
O-AEA_CSF	PEA	0.1431	0.3848	0.826607407
OEA_CSF	DEA	0.1427	0.3861	0.825034737
DEA_CSF	DEA	0.1576	0.3889	0.826667016
DH-g-LEA_CSF	AEA_CSF	0.1416	0.3899	0.824476042
bmi	Male sex	0.1388	0.3931	0.826935751
SEA_CSF	OEA_CSF	0.1405	0.3936	0.823719588
pain_sleep	AEA	0.1382	0.3949	0.822202051
pain_stress	AEA	0.1367	0.4002	0.828985714
AEA_CSF	pain_mood	-0.1385	0.4005	0.825395939
NADA_CSF	2_AGE_CSF	0.1383	0.4013	0.822867677
dvprs_movement	DEA	-0.1349	0.4066	0.829545729
PEA_CSF	SEA	-0.1347	0.4135	0.839405
NADA_CSF	dvprs_rest	0.1334	0.4181	0.844520398
DEA_CSF	dvprs_rest	0.1471	0.4218	0.847776238
DH-g-LEA_CSF	DEA	-0.1322	0.4226	0.8452
DH-g-LEA_CSF	AEA	-0.1321	0.4228	0.841454902
2_AG_CSF	dvprs_rest	0.1319	0.4236	0.838934634
O-AEA_CSF	pain_sleep	-0.1308	0.4274	0.842351456
DEA_CSF	age	-0.1451	0.4282	0.839851208
OEA_CSF	ODA_CSF	-0.1306	0.4282	0.835813462
OEA_CSF	Male sex	-0.1305	0.4285	0.832397129
age	LEA	0.1286	0.4292	0.829786667
OEA_CSF	pain_stress	0.1292	0.4331	0.833358294
SEA_CSF	2_AG_CSF	-0.1269	0.4413	0.845131132
AEA_CSF	pain_stress	-0.1263	0.4436	0.845547418

DH-g-LEA_CSF	pain_mood	-0.1263	0.4438	0.841975701
SEA_CSF	pain_sleep	-0.1259	0.4451	0.840514419
2_AGE_CSF	Male sex	0.1257	0.4459	0.838126852
LEA_CSF	bmi	0.1245	0.45	0.841935484
LEA_CSF	AEA_CSF	-0.122	0.4595	0.855766055
O-AEA_CSF	DEA_CSF	0.1353	0.4604	0.853526941
pain_activity	PEA	-0.1187	0.4658	0.859612727
PEA_CSF	NADA_CSF	0.1188	0.4712	0.865643439
DEA_CSF	pain_activity	-0.1311	0.4743	0.867413514
pain_stress	dvprs_rest	0.1157	0.4772	0.868803587
dvprs_movement	bmi	0.1149	0.4802	0.8703625
PEA_CSF	age	-0.1161	0.4814	0.868659556
LEA_CSF	DEA	-0.1142	0.4889	0.878289381
2_AG_CSF	DEA	-0.114	0.4897	0.875851101
dvprs_movement	OEA	-0.1121	0.4911	0.874502632
Male sex	AEA	-0.111	0.4953	0.878130131
ODA_CSF	PEA	-0.1119	0.4975	0.878195652
pain_mood	AEA	0.1102	0.4986	0.876327273
NADA_CSF	AEA_CSF	0.1114	0.4994	0.87395
2_AGE_CSF	DEA	-0.1111	0.5006	0.872290129
pain_mood	age	0.1096	0.5008	0.868909402
LEA_CSF	OEA	-0.1103	0.5037	0.870222128
AEA_CSF	1_2LG	0.1095	0.5069	0.872039831
pain_activity	age	-0.1078	0.508	0.870244726
pain_mood	LEA	0.1077	0.5081	0.866758824
SEA_CSF	age	0.109	0.5088	0.864321339
NADA_CSF	DEA_CSF	0.1206	0.5108	0.864103333
2_AG_CSF	2_AG	-0.1084	0.5111	0.861023237
pain_sleep	1_2LG	-0.1036	0.5245	0.879946281
DEA_CSF	2_AG_CSF	0.1166	0.5252	0.87749465
SEA_CSF	pain_activity	0.1047	0.5259	0.875063115
SEA_CSF	dvprs_movement	-0.1028	0.5336	0.884251429
pain_mood	DEA	0.1003	0.5379	0.887753659
OEA_CSF	2_AGE_CSF	0.1006	0.5423	0.891391903
OEA_CSF	2_AG	0.1002	0.5439	0.890416935
DH-g-LEA_CSF	pain_stress	-0.1002	0.5441	0.887167068
DH-g-LEA_CSF	2_AG_CSF	0.0993	0.5474	0.8889776
SEA	pain_activity	-0.0977	0.5488	0.887700398
DEA_CSF	SEA	-0.1093	0.5517	0.88885
O-AEA_CSF	1_2LG	0.0968	0.5579	0.895286166
AEA_CSF	Male sex	0.0967	0.5583	0.892400787
O-AEA_CSF	AEA	0.0956	0.5628	0.896065882
O-AEA_CSF	DEA	0.0939	0.5695	0.903191406
O-AEA_CSF	bmi	-0.0925	0.5753	0.908839689
O-AEA_CSF	dvprs_movement	-0.0919	0.5778	0.909251163
O-AEA_CSF	AEA_CSF	0.0912	0.5808	0.910443243
DEA_CSF	pain_sleep	0.1012	0.5814	0.907878462

2_AG_CSF	LEA	-0.0895	0.588	0.914666667
dvprs_rest	OEA	-0.0868	0.5943	0.920938168
PEA_CSF	OEA	-0.088	0.5944	0.917590875
DH-g-LEA_CSF	bmi	-0.0879	0.5945	0.914268939
O-AEA_CSF	DH-g-LEA_CSF	-0.0877	0.5955	0.912350943
LEA_CSF	PEA	-0.0877	0.5957	0.909226316
dvprs_movement	age	-0.085	0.602	0.915400749
pain_stress	LEA	0.0842	0.6053	0.916984328
ODA_CSF	age	-0.0843	0.6097	0.920216357
ODA_CSF	SEA	-0.084	0.6111	0.918913333
SEA_CSF	AEA_CSF	0.084	0.6111	0.915522509
NADA_CSF	2_AG	-0.0838	0.6118	0.913201471
DEA_CSF	bmi	0.0929	0.6132	0.911938462
NADA_CSF	age	-0.0833	0.614	0.90979562
AEA_CSF	OEA	0.0827	0.6167	0.910473455
PEA_CSF	pain_stress	-0.0826	0.617	0.907615942
pain_activity	2_AG	-0.0813	0.6178	0.905511913
SEA_CSF	NADA_CSF	-0.0806	0.6259	0.914084173
AEA_CSF	age	0.08	0.6284	0.914445878
pain_activity	bmi	0.0787	0.6293	0.912485
ODA_CSF	O-AEA_CSF	0.0789	0.6328	0.914294662
NADA_CSF	2_AG_CSF	-0.0775	0.639	0.919978723
ODA_CSF	DEA_CSF	0.0834	0.65	0.932508834
ODA_CSF	DEA	-0.0743	0.6531	0.933657042
DEA_CSF	1_2LG	0.0821	0.655	0.933087719
DEA_CSF	pain_mood	-0.0808	0.6602	0.937206993
ODA_CSF	Male sex	0.0725	0.661	0.935073171
pain_activity	dvprs_rest	0.0707	0.6646	0.936901389
OEA_CSF	pain_activity	-0.071	0.6675	0.937733564
PEA_CSF	bmi	-0.0709	0.6681	0.93534
SEA_CSF	PEA_CSF	-0.0706	0.6691	0.933520962
PEA_CSF	AEA	-0.0693	0.6749	0.938388356
DH-g-LEA_CSF	OEA	-0.0683	0.6795	0.941559727
O-AEA_CSF	Male sex	0.0677	0.6823	0.94222381
O-AEA_CSF	2_AG	-0.0676	0.6827	0.939580339
PEA_CSF	2_AG_CSF	0.067	0.6853	0.939972297
ODA_CSF	OEA	-0.0654	0.6925	0.946649832
DH-g-LEA_CSF	pain_sleep	0.0638	0.6994	0.952873826
DEA_CSF	dvprs_movement	0.0704	0.7019	0.953081605
2_AG_CSF	OEA	-0.063	0.7034	0.951934667
ODA_CSF	LEA_CSF	0.063	0.7034	0.948772093
SEA_CSF	2_AGE_CSF	-0.0626	0.7052	0.948050331
dvprs_movement	PEA	-0.0605	0.7106	0.952157096
DH-g-LEA_CSF	age	-0.0599	0.7173	0.957973026
PEA_CSF	dvprs_rest	0.0598	0.7174	0.954965246
PEA_CSF	dvprs_movement	-0.0594	0.7192	0.95423268
AEA_CSF	2_AG	-0.0594	0.7194	0.951388925

NADA_CSF	PEA	0.0589	0.7217	0.951331818
dvprs_movement	LEA	-0.0569	0.7271	0.95534822
DH-g-LEA_CSF	SEA	-0.0577	0.7271	0.952266452
DH-g-LEA_CSF	dvprs_movement	0.0573	0.7291	0.951815434
NADA_CSF	AEA	0.0568	0.7313	0.951627564
DH-g-LEA_CSF	Male sex	-0.0556	0.7367	0.955591693
AEA_CSF	dvprs_rest	-0.0542	0.743	0.960694268
PEA_CSF	2_AGE_CSF	0.0538	0.7448	0.959964444
pain_stress	2_AG	0.0528	0.746	0.958468354
SEA_CSF	DEA_CSF	-0.0563	0.7597	0.972991167
dvprs_rest	PEA	-0.0478	0.7698	0.982826415
2_AG_CSF	PEA	-0.048	0.7718	0.982290909
OEA_CSF	bmi	-0.0468	0.7773	0.986199375
NADA_CSF	pain_sleep	0.0468	0.7774	0.983253583
LEA_CSF	1_2LG	0.0466	0.7784	0.98146087
pain_stress	age	0.0457	0.7793	0.97955356
DEA_CSF	OEA	0.051	0.7818	0.979662963
AEA_CSF	2_AGE_CSF	0.0449	0.7859	0.981770462
ODA_CSF	pain_stress	-0.0449	0.786	0.978883436
AEA_CSF	LEA	0.0439	0.7906	0.981601223
PEA_CSF	2_AG	-0.0437	0.7917	0.979970122
LEA_CSF	age	0.0434	0.7929	0.97847234
dvprs_rest	bmi	0.041	0.8015	0.986087879
SEA_CSF	pain_stress	-0.0414	0.8025	0.984335347
SEA	dvprs_movement	-0.0402	0.8054	0.984916867
SEA	dvprs_rest	-0.0397	0.8077	0.984763363
PEA_CSF	LEA	-0.0401	0.8086	0.982908982
LEA_CSF	2_AGE_CSF	-0.0383	0.8171	0.990276418
SEA_CSF	pain_mood	0.0374	0.8213	0.992404167
dvprs_rest	LEA	0.0355	0.8277	0.997169733
LEA_CSF	AEA	-0.0343	0.8357	1
PEA_CSF	pain_mood	-0.0329	0.8425	1
LEA_CSF	dvprs_rest	0.0327	0.8434	1
SEA_CSF	O-AEA_CSF	0.0324	0.8448	1
ODA_CSF	LEA	-0.0318	0.8477	1
NADA_CSF	SEA	0.0313	0.8501	1
age	Male sex	-0.0301	0.8539	1
SEA_CSF	bmi	-0.0301	0.8558	1
LEA_CSF	LEA	-0.0296	0.8582	1
2_AG_CSF	1_2LG	0.0294	0.8592	1
NADA_CSF	OEA	0.0272	0.8693	1
LEA_CSF	pain_mood	0.0254	0.878	1
AEA_CSF	SEA	-0.0253	0.8785	1
OEA_CSF	pain_sleep	0.0245	0.8823	1
dvprs_rest	AEA	0.0241	0.8828	1
DH-g-LEA_CSF	pain_activity	-0.0243	0.8833	1
OEA_CSF	dvprs_movement	-0.0242	0.8837	1

2_AGE_CSF	bmi	0.0238	0.8857	1
DH-g-LEA_CSF	LEA	-0.0234	0.8876	1
2_AGE_CSF	pain_mood	-0.022	0.8944	1
2_AG_CSF	age	0.0204	0.9021	1
2_AG_CSF	SEA	0.0197	0.9051	1
DEA_CSF	PEA	-0.0192	0.9167	1
dvprs_rest	Male sex	0.0166	0.919	1
2_AGE_CSF	pain_stress	-0.0163	0.9214	1
LEA_CSF	DH-g-LEA_CSF	-0.0155	0.9254	1
AEA_CSF	PEA	0.0152	0.9269	1
PEA_CSF	pain_activity	-0.0151	0.9273	1
LEA_CSF	Male sex	-0.0145	0.9302	1
pain_mood	2_AG	0.0129	0.937	1
DEA_CSF	Male sex	0.0143	0.9383	1
SEA_CSF	LEA_CSF	-0.0123	0.9405	1
AEA_CSF	DEA	0.0119	0.9425	1
Male sex	1_2LG	-0.0116	0.9436	1
dvprs_movement	1_2LG	-0.0111	0.9459	1
OEA_CSF	NADA_CSF	-0.0111	0.9464	1
DH-g-LEA_CSF	2_AGE_CSF	-0.0102	0.9507	1
DEA_CSF	AEA	-0.0109	0.9527	1
LEA_CSF	SEA	-0.0097	0.9532	1
LEA_CSF	pain_stress	-0.0097	0.9534	1
NADA_CSF	Male sex	-0.0097	0.9534	1
O-AEA_CSF	NADA_CSF	0.0071	0.9659	1
AEA_CSF	bmi	-0.0061	0.9705	1
O-AEA_CSF	pain_stress	-0.0052	0.9749	1
ODA_CSF	AEA_CSF	-0.005	0.9761	1
NADA_CSF	LEA	0.0036	0.9824	1
O-AEA_CSF	dvprs_rest	-0.0032	0.9844	1
ODA_CSF	pain_sleep	0.0032	0.9847	1
bmi	2_AG	0.0027	0.9869	1
ODA_CSF	AEA	-0.0015	0.9927	1
dvprs_rest	DEA	0.0014	0.9929	1
bmi	1_2LG	0.0006	0.9972	1
2_AGE_CSF	1_2LG	-0.0002	0.999	1

Post Acetaminophen Correlations

Variable	by Variable	Spearman ρ	Prob>|ρ|	FDR Prob>|ρ|
AEA	1_2LG	0.6611	<0.0001	0.0025
DEA	1_2LG	0.6062	<.0001	0.0025
DEA	AEA	0.9418	<.0001	0.0025
LEA	1_2LG	0.6188	<.0001	0.0025
LEA	AEA	0.9358	<.0001	0.0025
LEA	DEA	0.8844	<.0001	0.0025
OEA	1_2LG	0.6911	<.0001	0.0025
OEA	AEA	0.9712	<.0001	0.0025
OEA	DEA	0.9205	<.0001	0.0025
OEA	LEA	0.9561	<.0001	0.0025
PEA	1_2LG	0.7442	<.0001	0.0025
PEA	AEA	0.9376	<.0001	0.0025
PEA	DEA	0.8771	<.0001	0.0025
PEA	LEA	0.8832	<.0001	0.0025
PEA	OEA	0.9517	<.0001	0.0025
SEA	1_2LG	0.7099	<.0001	0.0025
SEA	AEA	0.8639	<.0001	0.0025
SEA	DEA	0.7975	<.0001	0.0025
SEA	LEA	0.8512	<.0001	0.0025
SEA	OEA	0.8991	<.0001	0.0025
SEA	PEA	0.9385	<.0001	0.0025
ODA_CSF	1_2LG	-0.6489	<.0001	0.0025
ODA_CSF	OEA	-0.5869	<.0001	0.0025
ODA_CSF	PEA	-0.6112	<.0001	0.0025
ODA_CSF	SEA	-0.6002	<.0001	0.0025

POD1 Correlations

Variable	by Variable	Spearman ρ	Prob>|ρ|	FDR Prob>|ρ|
DEA	AEA	0.7832	<.0001	0.0406
LEA	AEA	0.8944	<.0001	0.0406
LEA	DEA	0.718	<.0001	0.0406
OEA	AEA	0.9191	<.0001	0.0406
OEA	DEA	0.7779	<.0001	0.0406
OEA	LEA	0.923	<.0001	0.0406
PEA	AEA	0.8802	<.0001	0.0406
PEA	DEA	0.7966	<.0001	0.0406
PEA	LEA	0.8934	<.0001	0.0406
PEA	OEA	0.9738	<.0001	0.0406
dvprs_movement	dvprs_rest	0.7926	<.0001	0.0406
pain_stress	pain_mood	0.7516	<.0001	0.0406
SEA	AEA	0.8172	<.0001	0.0406
SEA	DEA	0.8764	<.0001	0.0406
SEA	LEA	0.796	<.0001	0.0406
SEA	OEA	0.8757	<.0001	0.0406
SEA	PEA	0.9089	<.0001	0.0406
2_AG	1_2LG	0.5404	0.0005	0.1015
pain_mood	OEA	0.5345	0.0005	0.1015
pain_mood	AEA	0.5319	0.0006	0.0812
DH-g-LEA_CSF	PEA	-0.5337	0.0007	0.0711
pain_mood	LEA	0.5227	0.0008	0.0650
pain_sleep	pain_activity	0.5015	0.001	0.0677
PEA_CSF	DH-g-LEA_CSF	0.4874	0.0016	0.0928
pain_activity	dvprs_movement	0.4806	0.0017	0.0863
DH-g-LEA_CSF	SEA	-0.4946	0.0019	0.0857
pain_mood	DEA	0.4826	0.0022	0.0893
pain_sleep	dvprs_movement	0.4602	0.0028	0.1033

pain_mood	PEA	0.4721	0.0028	0.0947
ODA_CSF	PEA	-0.4767	0.0029	0.0906
pain_activity	dvprs_rest	0.4554	0.0032	0.0928
ODA_CSF	OEA	-0.4716	0.0032	0.0866
ODA_CSF	DH-g-LEA_CSF	0.4574	0.0034	0.0863
2_AG_CSF	2_AG	0.4596	0.0042	0.1003
DH-g-LEA_CSF	OEA	-0.4585	0.0043	0.0970
ODA_CSF	AEA	-0.4591	0.0043	0.0919
pain_sleep	DEA	0.4515	0.0044	0.0893
SEA_CSF	PEA	0.4531	0.0049	0.0947
SEA_CSF	OEA	0.4379	0.0067	0.1236
ODA_CSF	SEA	-0.4374	0.0068	0.1200
OEA_CSF	age	0.4215	0.0075	0.1269
DH-g-LEA_CSF	LEA	-0.4233	0.009	0.1462
NADA_CSF	DH-g-LEA_CSF	0.4106	0.0094	0.1468
NADA_CSF	pain_activity	-0.4055	0.0104	0.1564
SEA_CSF	DEA	0.4122	0.0112	0.1624
PEA_CSF	OEA_CSF	0.3901	0.0141	0.1974
pain_stress	dvprs_movement	0.3847	0.0143	0.1935
ODA_CSF	2_AG_CSF	-0.3864	0.0151	0.1978
OEA_CSF	LEA_CSF	0.386	0.0152	0.1929
pain_stress	LEA	0.3905	0.0154	0.1895
pain_mood	pain_sleep	0.3798	0.0156	0.1863
pain_sleep	dvprs_rest	0.3768	0.0165	0.1914
DH-g-LEA_CSF	AEA	-0.3898	0.0171	0.1929
PEA_CSF	DEA_CSF	0.4177	0.0174	0.1909
SEA_CSF	SEA	0.3791	0.0207	0.2212
ODA_CSF	DEA	-0.3782	0.021	0.2186
pain_stress	OEA	0.3723	0.0214	0.2172
PEA	2_AG	-0.3671	0.0234	0.2317
SEA_CSF	LEA	0.3701	0.0241	0.2330

SEA	pain_mood	0.3616	0.0257	0.2427
NADA_CSF	SEA	-0.3658	0.026	0.2399
SEA_CSF	AEA	0.3658	0.026	0.2346
pain_stress	AEA	0.3582	0.0273	0.2410
PEA_CSF	ODA_CSF	0.3494	0.0292	0.2522
pain_mood	dvprs_movement	0.3419	0.0308	0.2605
OEA_CSF	DEA_CSF	0.3814	0.0312	0.2585
OEA	2_AG	-0.3489	0.0318	0.2582
SEA	pain_sleep	0.3473	0.0327	0.2603
ODA_CSF	NADA_CSF	0.3399	0.0343	0.2678
ODA_CSF	LEA	-0.3477	0.035	0.2681
pain_stress	PEA	0.3373	0.0384	0.2887
age	2_AG	0.3243	0.047	0.3469
DH-g-LEA_CSF	DEA_CSF	0.3483	0.0508	0.3683
O-AEA_CSF	dvprs_rest	0.3135	0.052	0.3704
O-AEA_CSF	age	0.3074	0.057	0.3990
DH-g-LEA_CSF	2_AG	0.3143	0.0582	0.4005
LEA_CSF	pain_activity	0.3055	0.0586	0.3965
LEA	2_AG	-0.3072	0.0606	0.4033
PEA_CSF	1_2LG	0.3112	0.0608	0.3981
DH-g-LEA_CSF	DEA	-0.31	0.0619	0.3989
SEA_CSF	DH-g-LEA_CSF	-0.3011	0.0625	0.3965
NADA_CSF	pain_sleep	-0.2989	0.0645	0.4029
pain_sleep	OEA	0.3026	0.0648	0.3986
NADA_CSF	PEA	-0.3046	0.0668	0.4048
pain_mood	pain_activity	0.2913	0.0682	0.4072
2_AGE_CSF	pain_activity	-0.294	0.0693	0.4078
pain_stress	pain_sleep	0.285	0.0747	0.4333
pain_sleep	PEA	0.2903	0.077	0.4403
SEA_CSF	Male sex	-0.2851	0.0785	0.4427
pain_stress	pain_activity	0.2806	0.0795	0.4422

ODA_CSF	bmi	-0.2836	0.0802	0.4400
DEA_CSF	pain_activity	0.3131	0.081	0.4385
2_AGE_CSF	pain_mood	-0.2817	0.0823	0.4397
PEA_CSF	PEA	-0.2848	0.0875	0.4614
OEA_CSF	O-AEA_CSF	0.2769	0.0879	0.4575
pain_sleep	AEA	0.28	0.0886	0.4553
DEA_CSF	dvprs_rest	0.3057	0.0888	0.4507
SEA_CSF	pain_activity	0.2755	0.0896	0.4491
SEA_CSF	2_AG	-0.2815	0.0914	0.4525
pain_sleep	LEA	0.2731	0.0972	0.4755
DEA_CSF	AEA_CSF	0.2973	0.0985	0.4761
OEA_CSF	1_2LG	0.2744	0.1002	0.4786
DEA_CSF	1_2LG	0.296	0.1059	0.4999
DEA_CSF	DEA	0.2955	0.1066	0.4975
SEA_CSF	ODA_CSF	-0.2621	0.1069	0.4932
PEA_CSF	2_AG	0.2665	0.1109	0.5059
LEA_CSF	DEA_CSF	0.2869	0.1114	0.5025
NADA_CSF	DEA	-0.2658	0.1118	0.4988
bmi	age	-0.2538	0.1141	0.5035
pain_activity	DEA	0.2549	0.1225	0.5348
age	LEA	-0.2535	0.1246	0.5382
SEA_CSF	pain_stress	0.2486	0.127	0.5428
PEA_CSF	AEA	-0.255	0.1277	0.5401
pain_sleep	1_2LG	-0.2509	0.1286	0.5383
SEA_CSF	1_2LG	-0.2535	0.1301	0.5390
2_AG_CSF	1_2LG	0.2531	0.1307	0.5360
ODA_CSF	dvprs_rest	0.2455	0.1319	0.5355
PEA_CSF	OEA	-0.2523	0.132	0.5306
SEA_CSF	pain_mood	0.2438	0.1347	0.5362
pain_stress	DEA	0.2468	0.1353	0.5333
NADA_CSF	OEA	-0.2501	0.1354	0.5286

2_AG_CSF	Male sex	-0.2416	0.1383	0.5348
bmi	DEA	0.2415	0.1441	0.5519
O-AEA_CSF	dvprs_movement	0.2361	0.1478	0.5608
DEA_CSF	pain_sleep	0.2577	0.1545	0.5808
ODA_CSF	2_AGE_CSF	0.2291	0.1605	0.5978
2_AGE_CSF	2_AG_CSF	-0.2279	0.1628	0.6009
age	1_2LG	0.2302	0.1645	0.6017
OEA_CSF	2_AG	0.2311	0.1687	0.6115
DEA_CSF	2_AG	0.2531	0.1696	0.6094
OEA_CSF	2_AG_CSF	0.2221	0.1742	0.6204
O-AEA_CSF	LEA_CSF	0.2217	0.175	0.6178
PEA_CSF	LEA_CSF	0.2213	0.1759	0.6157
LEA_CSF	1_2LG	0.2223	0.1861	0.6458
age	DEA	-0.2184	0.1877	0.6458
NADA_CSF	LEA_CSF	-0.2148	0.1892	0.6455
OEA_CSF	DH-g-LEA_CSF	0.2144	0.19	0.6428
2_AG_CSF	bmi	0.2142	0.1903	0.6385
LEA_CSF	2_AG_CSF	0.2121	0.1948	0.6483
2_AGE_CSF	LEA	-0.2171	0.1968	0.6496
NADA_CSF	bmi	-0.2099	0.1997	0.6539
2_AGE_CSF	pain_stress	-0.2094	0.2007	0.6519
DEA_CSF	2_AGE_CSF	0.2319	0.2016	0.6496
dvprs_movement	DEA	0.211	0.2035	0.6506
OEA_CSF	pain_activity	0.205	0.2106	0.6680
O-AEA_CSF	2_AGE_CSF	0.2012	0.2193	0.6902
PEA_CSF	LEA	-0.2048	0.2241	0.6999
SEA	2_AG	-0.2002	0.2282	0.7072
SEA	pain_stress	0.1983	0.2327	0.7157
pain_activity	2_AG	-0.1971	0.2357	0.7195
NADA_CSF	LEA	-0.1973	0.2418	0.7326
PEA_CSF	SEA	-0.1963	0.2442	0.7344

PEA_CSF	O-AEA_CSF	-0.1903	0.2459	0.7341
LEA_CSF	pain_sleep	0.1884	0.2507	0.7430
2_AG_CSF	dvprs_rest	-0.1881	0.2514	0.7396
2_AG_CSF	pain_stress	-0.186	0.257	0.7507
PEA_CSF	AEA_CSF	0.1854	0.2584	0.7494
age	AEA	-0.1848	0.2666	0.7677
pain_sleep	bmi	0.1796	0.2675	0.7648
dvprs_movement	Male sex	0.1771	0.2744	0.7791
ODA_CSF	dvprs_movement	0.1792	0.275	0.7753
PEA_CSF	Male sex	-0.1788	0.2761	0.7731
2_AGE_CSF	OEA	-0.1828	0.2789	0.7756
2_AGE_CSF	age	0.1766	0.2822	0.7794
2_AGE_CSF	PEA	-0.1812	0.2832	0.7769
AEA_CSF	dvprs_rest	0.1756	0.2849	0.7763
2_AGE_CSF	2_AG	0.1777	0.2926	0.7920
OEA_CSF	DEA	0.1772	0.2941	0.7908
AEA	2_AG	-0.1737	0.2969	0.7930
PEA	age	-0.1737	0.2971	0.7884
pain_activity	1_2LG	-0.1721	0.3014	0.7946
SEA	dvprs_movement	0.172	0.3019	0.7908
PEA_CSF	pain_stress	-0.1684	0.3056	0.7953
AEA_CSF	2_AG_CSF	-0.1623	0.3237	0.8371
pain_activity	OEA	0.1644	0.3241	0.8328
DH-g-LEA_CSF	1_2LG	0.1661	0.326	0.8324
SEA	pain_activity	0.1632	0.3277	0.8315
age	OEA	-0.163	0.3282	0.8276
pain_stress	dvprs_rest	0.1578	0.3307	0.8288
OEA_CSF	pain_mood	0.1567	0.3407	0.8486
2_AGE_CSF	DEA	-0.1569	0.3538	0.8759
2_AG_CSF	DEA	0.1551	0.3593	0.8841
SEA_CSF	pain_sleep	0.1495	0.3636	0.8893

O-AEA_CSF	2_AG_CSF	-0.149	0.3653	0.8881
pain_activity	PEA	0.1499	0.3691	0.8920
AEA_CSF	pain_sleep	0.1475	0.3703	0.8896
OEA_CSF	AEA_CSF	0.1469	0.3723	0.8891
AEA_CSF	dvprs_movement	0.1465	0.3734	0.8866
DH-g-LEA_CSF	pain_activity	-0.1446	0.3797	0.8963
OEA_CSF	SEA	0.1465	0.3869	0.9080
pain_activity	Male sex	-0.1403	0.3878	0.9049
dvprs_movement	1_2LG	-0.1439	0.3886	0.9016
DH-g-LEA_CSF	AEA_CSF	0.1416	0.3899	0.8994
DEA	2_AG	-0.1431	0.3915	0.8980
bmi	Male sex	0.1388	0.3931	0.8966
SEA_CSF	OEA_CSF	0.1405	0.3936	0.8927
pain_sleep	2_AG	-0.1402	0.4013	0.9052
NADA_CSF	2_AGE_CSF	0.1383	0.4013	0.9002
pain_mood	2_AG	-0.139	0.4051	0.9037
LEA_CSF	DEA	0.1387	0.4129	0.9161
dvprs_movement	LEA	0.1352	0.4184	0.9232
2_AGE_CSF	AEA	-0.136	0.4222	0.9266
PEA_CSF	dvprs_rest	0.132	0.4233	0.9240
2_AG_CSF	dvprs_movement	-0.1316	0.4246	0.9219
pain_sleep	Male sex	0.1293	0.4267	0.9215
ODA_CSF	pain_mood	-0.1306	0.4281	0.9196
DEA_CSF	age	-0.1451	0.4282	0.9150
OEA_CSF	ODA_CSF	-0.1306	0.4282	0.9102
OEA_CSF	Male sex	-0.1305	0.4285	0.9061
OEA_CSF	OEA	0.1337	0.4301	0.9048
NADA_CSF	pain_stress	-0.1269	0.4413	0.9235
SEA_CSF	2_AG_CSF	-0.1269	0.4413	0.9188
PEA_CSF	pain_activity	0.1261	0.4444	0.9205
2_AGE_CSF	Male sex	0.1257	0.4459	0.9190

LEA_CSF	bmi	0.1245	0.45	0.9227
dvprs_rest	Male sex	0.1221	0.4529	0.9240
pain_stress	bmi	-0.1213	0.4558	0.9253
SEA_CSF	dvprs_rest	0.1229	0.456	0.9211
LEA_CSF	dvprs_movement	0.1221	0.459	0.9225
LEA_CSF	AEA_CSF	-0.122	0.4595	0.9190
O-AEA_CSF	DEA_CSF	0.1353	0.4604	0.9163
dvprs_movement	AEA	0.1226	0.4635	0.9180
OEA_CSF	AEA	0.1235	0.4663	0.9190
pain_stress	1_2LG	-0.1212	0.4684	0.9187
pain_mood	dvprs_rest	0.1178	0.4692	0.9158
2_AG_CSF	SEA	0.1226	0.4699	0.9128
NADA_CSF	AEA	-0.1224	0.4706	0.9098
2_AG_CSF	AEA	0.1222	0.4711	0.9065
PEA_CSF	NADA_CSF	0.1188	0.4712	0.9024
DEA	1_2LG	-0.1204	0.4716	0.8989
2_AGE_CSF	dvprs_rest	0.1182	0.4736	0.8985
O-AEA_CSF	2_AG	-0.1197	0.4804	0.9072
PEA_CSF	age	-0.1161	0.4814	0.9049
DH-g-LEA_CSF	pain_stress	-0.1156	0.4834	0.9044
AEA_CSF	2_AG	-0.1179	0.4869	0.9068
2_AG_CSF	pain_activity	0.1141	0.4892	0.9069
O-AEA_CSF	1_2LG	-0.1169	0.4908	0.9057
LEA	1_2LG	-0.1151	0.4912	0.9024
PEA_CSF	pain_mood	-0.1116	0.4987	0.9120
LEA_CSF	AEA	-0.1148	0.4988	0.9081
NADA_CSF	AEA_CSF	0.1114	0.4994	0.9052
pain_mood	Male sex	-0.1084	0.5053	0.9118
OEA_CSF	pain_sleep	0.1099	0.5054	0.9079
dvprs_rest	DEA	0.1111	0.5067	0.9063
SEA_CSF	age	0.109	0.5088	0.9060

PEA	bmi	0.1101	0.5105	0.9051
NADA_CSF	DEA_CSF	0.1206	0.5108	0.9017
NADA_CSF	1_2LG	0.1092	0.5201	0.9141
LEA_CSF	2_AG	0.1092	0.5202	0.9104
PEA_CSF	DEA	-0.1089	0.5211	0.9080
pain_stress	2_AG	-0.1066	0.5243	0.9097
DEA_CSF	2_AG_CSF	0.1166	0.5252	0.9074
O-AEA_CSF	pain_activity	0.1048	0.5256	0.9042
bmi	1_2LG	-0.105	0.5305	0.9088
OEA_CSF	dvprs_rest	0.1031	0.5323	0.9080
SEA_CSF	dvprs_movement	0.102	0.5366	0.9115
pain_mood	1_2LG	-0.1031	0.5381	0.9103
OEA_CSF	2_AGE_CSF	0.1006	0.5423	0.9136
DH-g-LEA_CSF	2_AG_CSF	0.0993	0.5474	0.9184
OEA	1_2LG	-0.0997	0.5515	0.9214
DEA_CSF	SEA	0.1101	0.5555	0.9243
SEA	bmi	0.0984	0.5567	0.9225
AEA_CSF	Male sex	0.0967	0.5583	0.9214
PEA	1_2LG	-0.0979	0.5589	0.9187
dvprs_rest	bmi	-0.0924	0.5707	0.9343
dvprs_rest	age	-0.0923	0.5713	0.9315
LEA_CSF	LEA	-0.0959	0.5723	0.9294
LEA_CSF	dvprs_rest	0.0931	0.5728	0.9265
O-AEA_CSF	bmi	-0.0925	0.5753	0.9269
O-AEA_CSF	AEA_CSF	0.0912	0.5808	0.9320
SEA	1_2LG	-0.0912	0.5863	0.9372
NADA_CSF	dvprs_movement	-0.0892	0.5894	0.9384
pain_activity	age	-0.0878	0.5902	0.9360
LEA_CSF	pain_mood	0.0883	0.5928	0.9365
DH-g-LEA_CSF	bmi	-0.0879	0.5945	0.9355
O-AEA_CSF	DH-g-LEA_CSF	-0.0877	0.5955	0.9335

dvprs_movement	OEA	0.088	0.5994	0.9360
dvprs_rest	1_2LG	-0.087	0.6033	0.9385
NADA_CSF	dvprs_rest	0.0856	0.6042	0.9363
LEA_CSF	pain_stress	0.085	0.6071	0.9372
ODA_CSF	age	-0.0843	0.6097	0.9376
SEA_CSF	AEA_CSF	0.084	0.6111	0.9363
O-AEA_CSF	pain_mood	0.0836	0.6127	0.9352
DEA_CSF	bmi	0.0929	0.6132	0.9324
NADA_CSF	age	-0.0833	0.614	0.9302
Male sex	OEA	-0.0835	0.6183	0.9332
dvprs_movement	age	-0.0808	0.6201	0.9324
DEA_CSF	AEA	0.0913	0.6253	0.9368
SEA_CSF	NADA_CSF	-0.0806	0.6259	0.9342
AEA_CSF	age	0.08	0.6284	0.9345
ODA_CSF	1_2LG	-0.0817	0.6308	0.9347
bmi	AEA	0.0801	0.6324	0.9337
ODA_CSF	O-AEA_CSF	0.0789	0.6328	0.9309
2_AGE_CSF	SEA	-0.0799	0.6383	0.9356
AEA_CSF	pain_mood	-0.0776	0.6388	0.9329
AEA_CSF	pain_activity	0.0775	0.639	0.9299
NADA_CSF	2_AG_CSF	-0.0775	0.639	0.9266
AEA	1_2LG	-0.0783	0.6405	0.9254
ODA_CSF	DEA_CSF	0.0834	0.65	0.9358
SEA	age	-0.0747	0.656	0.9411
ODA_CSF	Male sex	0.0725	0.661	0.9450
2_AGE_CSF	dvprs_movement	-0.0724	0.6613	0.9421
O-AEA_CSF	SEA	0.0735	0.6655	0.9447
PEA_CSF	bmi	-0.0709	0.6681	0.9451
SEA_CSF	PEA_CSF	-0.0706	0.6691	0.9432
dvprs_movement	PEA	0.0695	0.6785	0.9532
DEA_CSF	OEA	0.0772	0.6797	0.9516

pain_activity	LEA	0.0691	0.6802	0.9490
O-AEA_CSF	Male sex	0.0677	0.6823	0.9487
PEA_CSF	2_AG_CSF	0.067	0.6853	0.9496
DH-g-LEA_CSF	pain_mood	-0.0661	0.6894	0.9520
DEA_CSF	dvprs_movement	0.0727	0.6924	0.9529
2_AGE_CSF	pain_sleep	-0.0654	0.6926	0.9500
Male sex	1_2LG	-0.0658	0.6949	0.9499
LEA_CSF	SEA	0.0657	0.6994	0.9529
ODA_CSF	LEA_CSF	0.063	0.7034	0.9551
pain_activity	AEA	0.0635	0.7048	0.9538
SEA_CSF	2_AGE_CSF	-0.0626	0.7052	0.9512
Male sex	LEA	-0.0632	0.706	0.9491
DH-g-LEA_CSF	age	-0.0599	0.7173	0.9611
OEA_CSF	PEA	0.0612	0.719	0.9602
bmi	OEA	0.0583	0.7279	0.9689
pain_stress	Male sex	-0.0558	0.7321	0.9713
pain_mood	age	-0.0554	0.7344	0.9712
bmi	2_AG	-0.0565	0.7363	0.9706
DH-g-LEA_CSF	Male sex	-0.0556	0.7367	0.9680
PEA	Male sex	-0.0557	0.74	0.9692
pain_mood	bmi	-0.054	0.7405	0.9667
PEA_CSF	2_AGE_CSF	0.0538	0.7448	0.9692
DEA_CSF	LEA	0.0603	0.7473	0.9693
AEA_CSF	OEA	0.0529	0.756	0.9775
pain_stress	age	0.0501	0.7588	0.9780
SEA_CSF	DEA_CSF	-0.0563	0.7597	0.9761
bmi	LEA	-0.051	0.7612	0.9749
AEA_CSF	DEA	0.0488	0.7745	0.9888
2_AG_CSF	OEA	0.0486	0.7751	0.9865
dvprs_rest	LEA	0.0477	0.7762	0.9848
OEA_CSF	bmi	-0.0468	0.7773	0.9831

pain_sleep	age	-0.0458	0.779	0.9822
AEA_CSF	2_AGE_CSF	0.0449	0.7859	0.9878
LEA_CSF	age	0.0434	0.7929	0.9936
O-AEA_CSF	AEA	-0.0441	0.7955	0.9938
PEA_CSF	pain_sleep	-0.0425	0.7975	0.9932
SEA	Male sex	-0.043	0.7977	0.9904
DEA_CSF	pain_stress	-0.0455	0.8046	0.9959
SEA	dvprs_rest	0.0409	0.8076	0.9966
AEA_CSF	pain_stress	0.0399	0.8093	0.9957
O-AEA_CSF	OEA	0.0408	0.8106	0.9943
LEA_CSF	2_AGE_CSF	-0.0383	0.8171	0.9992
2_AG_CSF	pain_sleep	-0.038	0.8182	0.9976
Male sex	AEA	-0.0379	0.8211	0.9981
LEA_CSF	OEA	-0.0377	0.8247	0.9995
PEA_CSF	dvprs_movement	-0.0366	0.8248	0.9966
AEA_CSF	1_2LG	0.0369	0.8285	0.9981
dvprs_rest	AEA	0.0353	0.8333	1
dvprs_rest	PEA	-0.0347	0.8361	1
Male sex	DEA	0.0329	0.8444	1
SEA_CSF	O-AEA_CSF	0.0324	0.8448	1
O-AEA_CSF	PEA	0.0328	0.847	1
DEA_CSF	pain_mood	-0.0344	0.8515	1
age	Male sex	-0.0301	0.8539	1
SEA_CSF	bmi	-0.0301	0.8558	1
O-AEA_CSF	pain_stress	0.0293	0.8593	1
DH-g-LEA_CSF	dvprs_movement	-0.0282	0.8646	1
AEA_CSF	AEA	-0.0288	0.8658	1
dvprs_movement	bmi	-0.0266	0.8707	1
2_AG_CSF	PEA	0.0256	0.8804	1
OEA_CSF	pain_stress	0.0246	0.8817	1
O-AEA_CSF	pain_sleep	0.0246	0.8819	1

ODA_CSF	pain_stress	-0.0241	0.8842	1
2_AGE_CSF	bmi	0.0238	0.8857	1
O-AEA_CSF	DEA	-0.0238	0.889	1
pain_activity	bmi	-0.0225	0.8904	1
ODA_CSF	2_AG	-0.0225	0.8946	1
dvprs_rest	2_AG	-0.021	0.9004	1
AEA_CSF	PEA	-0.0211	0.9013	1
2_AG_CSF	age	0.0204	0.9021	1
NADA_CSF	pain_mood	-0.0199	0.9041	1
2_AG_CSF	LEA	-0.0196	0.9085	1
dvprs_movement	2_AG	-0.019	0.91	1
OEA_CSF	LEA	-0.0172	0.9196	1
LEA_CSF	DH-g-LEA_CSF	-0.0155	0.9254	1
Male sex	2_AG	-0.0152	0.9279	1
LEA_CSF	Male sex	-0.0145	0.9302	1
ODA_CSF	pain_sleep	0.0145	0.9302	1
DH-g-LEA_CSF	dvprs_rest	-0.0141	0.932	1
DEA_CSF	Male sex	0.0143	0.9383	1
SEA_CSF	LEA_CSF	-0.0123	0.9405	1
OEA_CSF	NADA_CSF	-0.0111	0.9464	1
AEA_CSF	LEA	-0.011	0.9484	1
DH-g-LEA_CSF	2_AGE_CSF	-0.0102	0.9507	1
OEA_CSF	dvprs_movement	-0.0101	0.9513	1
2_AGE_CSF	1_2LG	-0.0102	0.9522	1
NADA_CSF	Male sex	-0.0097	0.9534	1
ODA_CSF	pain_activity	-0.0095	0.9541	1
LEA_CSF	PEA	0.0087	0.9594	1
DEA_CSF	PEA	0.0093	0.9605	1
O-AEA_CSF	LEA	-0.0084	0.9606	1
O-AEA_CSF	NADA_CSF	0.0071	0.9659	1
DH-g-LEA_CSF	pain_sleep	0.0067	0.9678	1

AEA_CSF	bmi	-0.0061	0.9705	1
ODA_CSF	AEA_CSF	-0.005	0.9761	1
NADA_CSF	2_AG	-0.0047	0.9778	1
dvprs_rest	OEA	-0.0042	0.9799	1
AEA_CSF	SEA	0.0037	0.9828	1
2_AG_CSF	pain_mood	-0.0024	0.9887	1
